# Subcutaneous inoculation of *Escherichia coli* in broiler chickens causes cellulitis and elicits innate and specific immune responses

**DOI:** 10.1186/s12917-024-04392-2

**Published:** 2024-12-02

**Authors:** Liv Jonare, Eva Wattrang, Emma Östlund, Helena Wall, Magdalena Jacobson, Désirée S. Jansson

**Affiliations:** 1 Department of Clinical Sciences, Swedish University of Agricultural Sciences, Box 7054, 750 07 Uppsala, Sweden; 2 Department of Microbiology, Swedish Veterinary Agency, 751 89 Uppsala, Sweden; 3 Department of Applied Animal Science and Welfare, Swedish University of Agricultural Sciences, Box 7024, 750 07 Uppsala, Sweden

**Keywords:** Cellulitis, APEC, Chicken mannose receptor MRC1L-B, Immune response, *Post-mortem* findings, IgY, Clinical signs

## Abstract

**Background:**

Cellulitis caused by *Escherichia coli* is a common cause of condemnation of broiler chickens at slaughter worldwide and is associated with economic losses and a possible negative impact on animal welfare. The study objective was to monitor clinical signs and immune responses after subcutaneous *E. coli* inoculation (1.1–1.8 × 10^7^ CFU), aiming to induce cellulitis. Three groups of broiler chickens (*n* = 15/group) were inoculated with well-characterized *E. coli* strains (group A: ECA18 O24:H4/ST117 and group B: ECB11 O153:H9/ST38) or with saline (control) at 22 days-of-age. Clinical signs of disease, body weight and immune parameters were monitored until euthanasia 12–14 days after inoculation followed by *post-mortem* examination.

**Results:**

The daily weight gain of the inoculated chickens was significantly lower one day after inoculation compared to the controls. Seven (23%) of the inoculated chickens displayed clinical signs: ruffled feathers, mild weakness, open-beak breathing and/or reluctance to stand, of which two birds were euthanized and one bird died. Five chickens in group B were observed with bacteraemia, which lasted up to three days after inoculation for two chickens. A transient increase in chicken mannose receptor MRC1L-B expression on circulating monocytes was observed one day after inoculation in both *E. coli* inoculated groups, with a more pronounced increase in group B. On day 7 after inoculation, the in vitro adherence of heterophils, monocytes and thrombocytes to the inoculated strain was increased in group B. Antibody titers to the inoculation strains were increased in some chickens in both groups on days 7 and 14 after inoculation, with the highest titers in group B. Seven (47%) and 13 (87%) of the chickens in group A and B, respectively, were diagnosed with cellulitis at *post-mortem* examination. In most birds, lesions consisted of plaque-like material embedded in the subcutaneous tissue of the abdominal wall.

**Conclusions:**

Inoculation of *E. coli* caused cellulitis and prompted a rapid activation/redistribution of circulating monocytes followed by antibody production. The responses were most pronounced in chickens inoculated with *E. coli* strain ECB11, presumably because of a higher virulence.

**Supplementary Information:**

The online version contains supplementary material available at 10.1186/s12917-024-04392-2.

## Background

Cellulitis in broiler chickens caused by avian pathogenic *Escherichia coli* (APEC) is a common cause of condemnation at slaughterhouses worldwide. In Europe, the condemnation rate due to skin lesions, of which cellulitis is presumed to be the most common finding, has been reported to vary between countries, 0.09–0.67% [1]. It can however be difficult to compare condemnation rates between countries due to different systems of condemnations codes applied at both national and regional levels [1]. Cellulitis results in economic losses for the broiler production chain and may have a negative impact on animal welfare [2]. The disease is characterized by a subcutaneous, serosanguineous to caseated fibrinous plaque, often covered by thickened and discoloured skin. The lesion is commonly located on the abdomen or in the vent region, but other areas of the body can also be affected such as the breast. Avian pathogenic *E. coli* is believed to enter the subcutaneous tissue through scratches or other skin lesions [3, 4]. Affected broiler chickens show few or no clinical signs [5, 6], and there are no diagnostic tools available to diagnose cellulitis in live birds on a farm. Therefore, features of the disease have instead been studied in experimental settings [4, 7–9]. These studies have reported body weight loss and skin lesions, *i.e.* oedema and discoloration, but no clinical signs of depression [7, 9]. Moreover, 54–100% of the birds developed cellulitis, which could be associated with *E. coli* isolate and inoculation dose. In contrast, mild to severe clinical signs of depression and high mortality rates have been described in experimental studies of other types of colibacillosis (*E. coli* associated infections), induced by *e.g.* the oviductal or air sac route [10–12]. The severity of the clinical signs was reported to correspond to the extent of gross organ lesion scoring *e.g.* fibrinopurulent peritonitis and oophoritis [10]. Further, it is somewhat unclear when broiler chickens develop cellulitis during grow-out on commercial farms. Johnson et al. showed that broilers could develop cellulitis at any age after experimental inoculation [13]. However, in birds over 16 days of age, cellulitis was not accompanied by signs such as pericarditis, perihepatitis and airsacculitis, which in contrast were common findings in younger birds. In another study on chickens condemned due to cellulitis at slaughter, the majority of birds with cellulitis had no other gross findings suggestive of *E. coli* infection, indicating that these birds had developed the disease in a mid to late grow-out phase [14].

Little is known about immune responses during the development of cellulitis caused by *E. coli* in broiler chickens. However, experimental *E. coli* infections of chickens have shown that epithelial barrier and soluble mediators such as host defence peptides, act as a first line of defence together with a rapid cellular response [15]. The cellular response is dominated by heterophils and macrophages, which are recruited by chemokines to the site of infection, where they pursue phagocytic, bactericidal and immunomodulatory activities [15, 16]. In addition, thrombocytes have been found to phagocytose bacteria, although to a lesser extent than heterophils [17]. Olkowski et al*.* investigated the first line of defence in cellulitis and showed that fewer heterophils and macrophages with lower phagocytic activity were recruited in broiler chickens, as compared to leghorn chickens of the same age, suggesting that broilers may be more susceptible to cellulitis caused by *E. coli* [18]. Moreover, a significant increase in antibody titers to *E. coli* was observed after experimental infection of non-vaccinated broiler breeders [19], and studies of the adaptive immune response have shown that antibodies may opsonise *E. coli*, which could increase bacterial clearance [11].

More knowledge on cellulitis development and the immune response is needed to further understand the pathogenesis, and in a wider perspective, decrease the occurrence of cellulitis. Thus, the aims of the present study were to monitor the clinical outcome, selected parameters of the immune response, and the gross findings in broiler chickens after subcutaneous *E. coli* inoculation.

## Materials and methods

### Bacterial strains and preparation of inocula

The two strains of *E. coli* used for inoculation (ECA18 and ECB11) had been isolated from cellulitis in two commercial broiler chickens condemned at slaughter and from two different flocks in 2020. Strain ECA18 originated from a chicken with local lesions on the abdomen and medial thigh (3 × 1 and 1 × 1 cm, respectively), with no gross evidence of systemic spread. Strain ECB11 originated from a chicken with a lesion extending from the abdomen to the thigh and breast (6 × 7 and 3 × 4 cm), and with pericarditis, hepatomegaly and peritonitis. The strains were stored in Brain Heart Infusion broth (BHI) supplemented with 15% glycerol (Swedish Veterinary Agency [SVA], Sweden) in −70 °C. Bacteria from the frozen stock were passaged twice on 5% bovine blood agar (SVA). One *E. coli* colony from each strain, as confirmed by matrix-assisted laser desorption ionization-time of flight mass spectrometry (MALDI-TOF MS; Maldi Biotyper Microflex LT System with the MBT BDAL 8468 MSP library, Bruker Daltonik GmbH), was placed in 10 mL of BHI and incubated for 18 h during shaking (37 °C, 80 rpm) to reach a concentration of 10^9^ CFU/mL. After incubation, the purity of the cultures was confirmed by phase-contrast microscopy. The bacteria were washed once in sterile 0.9% NaCl (B. Braun, Germany) and diluted 1:200. The final inocula were stored on ice and used for inoculation within 3.5 h. To confirm purity and inoculation dose, serial tenfold dilutions were cultured on 5% bovine blood agar, incubated at 37 °C over-night and the colonies were counted.

### Phenotypic antimicrobial susceptibility

Strain ECA18 and ECB11 were cultured on 5% bovine blood agar and passaged twice. Minimum inhibitory concentrations (MICs) were analysed at the Department of Animal Health and Antimicrobial Strategies, SVA following standards for microdilution of the Clinical and Laboratory Standards Institute [20] for amoxicillin-clavulanic acid, ampicillin, cefotaxime, cefalexin, colistin, enrofloxacin, gentamicin, meropenem, neomycin, nitrofurantoin, tetracycline, and trimethoprim-sulfamethoxazole. The *E. coli* strain ATCC25922 (accession number CP009072.1) was used as control. The MICs were compared to clinical breakpoints provided by EUCAST [21].

### Whole-genome sequencing, bioinformatics

Strains (ECA18 and ECB11) and a random selection of re-isolated pure cultures of *E. coli* that represented cellulitis lesions (*n* = 2) and blood (*n* = 3) obtained during the study, were whole-genome sequenced (WGS). Because the original strain ECB11 used for inoculation showed a phenotypic variation in colony morphology when retrieved from frozen stock, *i.e.* colonies were either light grey (ECB11a) or slightly darker grey (ECB11b), colonies of both phenotypes were sequenced. To prepare DNA, following two passages, bacteria were lysed by heating at 100 °C for 5 min. Nextera chemistry was used for library preparation. For the ECB11 sample, 2 × 300 bp reads were produced on an Illumina MiSeq instrument at SVA and for the other samples, 2 × 150 bp reads were produced on an Illumina NovaSeq instrument at SciLifeLab Clinical Genomics (Solna, Sweden). Reads were trimmed with Trimmomatic v. 0.39 [22] and downsampled to approximately 100 × coverage with the reformat script available in the BBMap v. 38.79 package [23]. Assemblies were created with Unicycler v. 0.4.8 [24]. Virulence genes and resistance genes were detected in the assemblies with VirulenceFinder v. 2.0.3 [25, 26] and ResFinder v. 4.1.11 [27–29], and the serotype was determined with SerotypeFinder v. 2.0.1 [30]. Multi-locus sequence type was determined with FastMLST v. 0.0.15 [31]. The trimmed and downsampled reads were mapped to the reference genome of *E. coli* strain K-12 MG1655 (accession number NC_000913.3) with Bowtie2 v. 2.4.5. Single nucleotide polymorphisms (SNPs) were called and filtered with BCFtools v. 1.14 [32] and an in-house Python script [33]. The two variants ECB11a and ECB11b were aligned with Mauve v. 2.4.0 [34]. The assembly of ECB11a was analysed with PHASTER (accessed 2022–10–19) [35, 36].

### Animals and housing

The study was conducted at the Swedish Livestock Research Centre of the Swedish University of Agricultural Sciences. Fifty non-sexed, one-day-old Ross 308 broiler chickens were purchased from a commercial hatchery (SweHatch AB, Sweden). The chickens were housed on wood shavings in ten raised pens measuring 0.75 × 1.5 m, with five randomly allocated chickens in each pen. The temperature was 31 °C on arrival, and gradually decreased to 18 °C on day 30, according to industry routines. The relative humidity ranged from 38–72%. Artificial light was set to 24 h/d during the first day and reduced with 1 or 2 h/day until 8 h of darkness was achieved (9 pm–1 am and 3 am–7 am), and natural light was provided during the day. A commercial feed (crude protein 215 g/kg, metabolizable energy 11.6 MJ/kg, lysine 13.5 g/kg, methionine 5.2 g/kg), without antimicrobials was given ad libitum on trays during the first week, and thereafter in feeding containers. Water was provided ad libitum in water nipples*.*

### Experimental design

The inoculation model used was adapted from previous studies that have induced typical cellulitis lesions at the inoculation site in the majority of birds [4, 7, 8]. The two *E*. *coli* strains used in this study were confirmed to induce cellulitis lesions in a pilot study, when chickens were necropsied 3–6 days post inoculation (PI; unpublished results, L. Jonare). All chickens were allowed to acclimatize for 14 days. A live attenuated vaccine against coccidiosis (Paracox®−5 vet., Intervet, Netherlands) was sprayed on the feed according to the manufacturer’s instructions at three days of age. Three pens with five chickens each were allocated to the respective experimental treatment group (inoculation groups A, B, and control C; 15 chickens/group; non-blinded design). The number of chickens included was decided based on power calculations of selected immune parameters described in Wattrang et al. [37]. These calculations indicated that a minimum of five chickens was needed to observe statistically significant effects in a systemic bacterial infection of chickens. As the current *E. coli* inoculation was predicted to mainly induce a local infection, more subtle effects on immune parameters were predicted. Therefore, a minimum of seven chickens per sampling group was chosen for the present study. At 22 days of age (experimental day 0), the chickens in groups A and B were inoculated subcutaneously on the right side of the ventral abdomen (0.5 mL). The inoculation doses were 1.1 × 10^7^ CFU/chicken (strain ECA18) in group A, and 1.8 × 10^7^ CFU/chicken in group B (strain ECB11). Control birds were sham-inoculated (sterile physiological 0.9% NaCl, 0.5 mL/chicken). The chickens were weighed on days 7 and 3 before inoculation, daily on 0 to 5 days PI, on days 7 and 10 PI and 12–14 days PI (after euthanasia). Twice daily throughout the experiment, bird health was assessed by observation. Birds displaying abnormalities were removed from the pens and examined individually including assessment of the general condition, body posture, activity level, crop contents, eyes, breathing, plumage and skin condition. In addition, thorough individual clinical examination, as described above, was performed on all chickens in the afternoon on day 4 before inoculation and on days 1–3, 5, 7, 10, and 12–14 PI, and signs of cellulitis were recorded. Chickens were euthanized if anomalies were observed (more than three mild or one severe). The assessment criteria were based on principles required by the Ethics committee.

Blood sampling was performed in the morning 4 or 3 days before inoculation (half of the chickens/day, from now on designated day −4) and on 1–4, and 7 days PI. For sampling on days 1–4 PI, each group was divided into two subgroups (*n* = 7 or 8), which were sampled on every other occasion to reduce the number of samplings per chicken. All chickens were sampled on experimental days −4 before inoculation and at 7 d PI. The control group was sampled first, followed by group A and then group B. Blood was drawn from the jugular vein and approximately 350 μL were added to a sterile tube supplemented with lithium heparin (35 IU/mL blood; S-Monovette® 1.2 mL, Sarstedt, Germany). The remaining blood was transferred to a microtube without additives (Axygen, United States). On days 12–14 PI (from now on designated day 14 PI), four to six chickens from each group were euthanized per day by intravenous pentobarbital injection (Allfatal vet., Omnidea, Sweden, 1 mL/kg). In addition, blood for serology only was collected *post-mortem* from the femoral vein in a microtube (Axygen).

### Post-mortem examination and re-isolation of* E. coli*

Sex, body condition, and presence of skin- and subcutaneous lesions were documented at *post-mortem* examination. All organs were assessed for findings indicative of bacterial infection. Hip, knee and hock joints, the femoral head and the tendon sheath above the hock, were assessed for signs of infection. Samples for bacterial culture were obtained from the inoculation site, the pericardium and the spleen parenchyma using Amies swabs (Copan, Italy) and aseptic technique. Swabs were cultured on 5% bovine blood agar and incubated at 37 °C over-night. For species confirmation, one representative colony from each culture was analysed by MALDI-TOF MS, passaged once on 5% bovine blood agar, and stored in BHI supplemented with 15% glycerol (SVA) in −70 °C, pending further analyses. For quantification of *E. coli* colonies in blood, serial tenfold dilutions of heparin-stabilized blood from individual chickens from each sampling occasion were inoculated on 5% bovine blood agar and incubated, confirmed and stored as described above. One sample from the gizzard and the caecal tonsils, respectively, was taken from five randomly selected birds (control group *n* = 2, A *n* = 1, B *n* = 2) and were analysed for Fowl Adenovirus (FAdV) by routine PCR methodology at SVA [38].

### Blood leukocyte counts

Absolute counts of different leukocyte populations were determined using a previously described no-lyse, no-wash, flow cytometry-based method [39]. Two panels of monoclonal antibodies were used (Table 1).

**Table 1:** Combinations of monoclonal antibodies (panels) used for immunolabelling of chicken leukocytes in flow cytometry

Abbreviation	Clone	Panel 1	Panel 2	Panel 3	Target
CD45-PerCPCy5.5	UM16-6^a c^	x		x	Chicken CD45, all isoforms [40]
CD41/61-FITC ^a^	11C3	x	x	x	Chicken integrin CD41/61 (GPIIb-IIIa)
Kul01-PE^b^	KUL01	x		x	Chicken mannose receptor MRC1L-B [41]
Bu-1-pacific blue^b^	AV20	x			Chicken Bu-1 (ChB6)
CD8β-PE^b^	EP42		x		Chicken CD8β
CD8ɑ-Cy5^b^	3–298		x		Chicken CD8ɑ
CD4-pacific blue^b^	CT-4		x		Chicken CD4
TCR1-PerCPCy5.5	TCR-1^a c^		x		Chicken TCR γ/δ
CD25-PECy5	AV142^a c^		x		Chicken IL-2 receptor ɑ chain

^a^Purchased from Bio-Rad

^b^Purchased from Southern BioTech

^c^Conjugated with Lightning Link® kits (Abcam)

Heterophils, monocytes, thrombocytes, lymphocytes and subpopulations of lymphocytes, *i.e.* B-cells, CD4+CD8-, CD4+CD8α + , CD4-CD8αα+ , CD4-CD8αα+ , TCR𝛾/𝛿+CD8-, TCR𝛾/𝛿 +CD8αα+ , and TCR𝛾/𝛿+CD8𝛼β+ cells, were identified, and CD25 expression on CD4+CD8-, CD4+CD8𝛼+ , CD4-CD8𝛼𝛼+ , CD4-CD8αα+ and TCR𝛾/𝛿+CD8-, TCR𝛾/𝛿+CD8αα+ and TCR𝛾/𝛿+CD8𝛼β+ cells was analysed as described in the gating strategies in Additional file S1 and S2. Samples were recorded for 1 min for panel 1 and 1.5 min for panel 2 at reduced flow rate (corresponding to approximately 15,000 events in the CD45+ gate in panel 1, and approximately 6500 events in the lymphocyte gate in panel 2, respectively) in a BD FACSVerse™ Flow cytometer (BD Bioscienes) equipped with 488 nm blue, 633 nm red and 405 nm violet UV lasers. The results were analysed using the FACSDiva software version 9.0 (BD Biosciences). Single-stained compensation controls and fluorescence-minus-one (FMO) negative controls were included in all flow cytometry protocols, and titrations of all antibodies used were performed to determine optimal labelling conditions prior to the experiment. The number of events counted in the bead gate was used to determine the volume of the blood sample analysed and to calculate absolute numbers of the leukocyte populations. Fluorescent beads 123 count eBeads (#01–1234-42, Invitrogen, Thermo Fisher Scientific, United States) were used.

### *E. coli *leukocyte adhesion assay

Adhesion of heterophils, monocytes and thrombocytes to fluorescent-labelled *E. coli* was assessed using a modified protocol based on an assay for phagocytosis in chicken whole-blood samples [42]. The CellTrace™ Far Red Cell Proliferation kit (#C34564, Invitrogen™, Thermo Fischer Scientific) was used for fluorescence labelling of *E. coli* strains ECA18 and ECB11 as described earlier [43], with modifications. Strains were retrieved from frozen stock, passaged twice on 5% bovine blood agar at 37 °C for 24 h, and were inoculated in BHI (SVA) for over-night culture at 37 °C. Following pelleting at 3220 × g for 10 min, the supernatant was removed and the bacteria were resuspended in 300 µL 5 mM working-dilution of Far Red dye per estimated 10^8^ CFU *E. coli*. The suspensions were incubated at 37 °C on a shaker in the dark for 20 min. Five-times the staining volume of RPMI 1640 medium (HyClone, Cytiva, United States) supplemented with 5% foetal bovine serum (Gibco™, Thermo Fisher Scientific) was added and the suspension was incubated at room temperature for 5 min. Bacteria were pelleted at 3220 × g for 10 min, the supernatant was removed and the bacteria were resuspended in RPMI without additives and stored at 4 °C in the dark until use. The concentrations (CFU/mL) of labelled *E. coli* for strain ECA18 and ECB11 were determined by tenfold serial dilutions of bacteria spread on 5% bovine blood agar plates and cultured for 24 h at 37 °C. For the *E. coli* adhesion assay, round bottom, anti-stick 96-well cell culture plates (#83.3925.500, TC plates susp R, Sarstedt, Germany) were used. To each well, 40 μL RPMI supplemented with 2 mM L-glutamine, 50 μL of panel 3 of fluorochrome-conjugated antibodies (Table 1) diluted in RPMI with L-glutamine, 25 μL of Far Red labelled *E. coli* diluted in RPMI with L-glutamine to 10^8^ CFU/mL, and 10 μL heparinised blood were added. The assay was performed on all blood samples using both strains. Plates were incubated at 40 °C for 15 min, 15 µL of 20 mM EDTA in phosphate buffered saline (PBS) was added to each well to a final concentration of 2 mM EDTA, and plates were subsequently incubated at room temperature for 10 min. Thereafter, 30 µL of 4% paraformaldehyde (#43,368, Alfa Aesar, Thermo Scientific) in PBS was added and mixed by repeated pipetting. The content of the wells was transferred to test tubes containing 300 µL FACS buffer (PBS supplemented with 0.2% bovine serum albumin, 0.2% sodium azide, 0.05% normal horse serum [Sigma-Aldrich] and 2 mM EDTA). Samples were then stored at 4 °C for a maximum of 3 days before analysis in a FACSVerse™ Flow cytometer (BD Biosciences), and the results were analysed using the FACSDiva software version 9.0 (BD Biosciences) as described above. The gating strategy to define different leukocyte populations and proportions of adhered *E. coli* is shown in Additional file S3.

### Quantification of IgY antibodies to *E. coli*

An in-house ELISA for quantification of IgY to *E. coli* in serum samples was set up by adapting a previously used protocol [39]. In brief, a sonicated preparation of either *E. coli* strain ECA18 or ECB11 was used as coating antigens at a final protein concentration of 1 μg/mL. The ELISA protocol was performed with horseradish peroxidase-conjugated polyclonal goat-anti-chicken IgG (IgY) Fc antibodies (#AAI29P, BioRad Antibodies, United States) as tracer, and a commercial substrate buffer (Svanovir substrate, Boehringer-Ingelheim, Germany) was used for visualisation of antibody-binding. Serum samples were titrated in twofold steps starting at dilutions 1:50 to 1:1000, depending on antibody concentration, to achieve a dilution curve. The A_450_ value for each sample was plotted against the dilution, and the equation for the linear part of the curve was determined by regression analysis. The antibody titres were calculated as the dilution factor if A_450_ = 1. Positive and negative control samples (with high and low antibody titer, respectively) were included on each plate. All samples were tested twice, *i.e.* once with each antigen.

### Statistics

Descriptive statistics and regression analysis was done in Microsoft Excel 2016 (Microsoft Corporation) and all chickens in the experimental groups A, B and controls were included in the analysis (*n* = 45). Numerical data for body weight, leukocyte counts, bacterial adhesion to leukocytes, and antibody titers were analysed as mean values ± 95% confidence intervals (CI), and mean values with non-overlapping CI were treated as rejecting the null hypothesis of no difference. For antibody titers geometrical mean values were calculated, and for all other data, arithmetic mean values were used. Significant associations to infection strains were analysed with Fishers Exact Test and correlations between antibody titers and individual adherence data used the Spearman’s rank correlation coefficient test with square-root transformed antibody titers. Geometric mean values, CI for geometric mean values, Fisher Exact Test and Spearman’s rank correlation coefficient test were calculated using the software RStudio 2021.09 Build 351 "Ghost Orchid" Release for Windows.

## Results

### Clinical signs

**Fig. 1:**
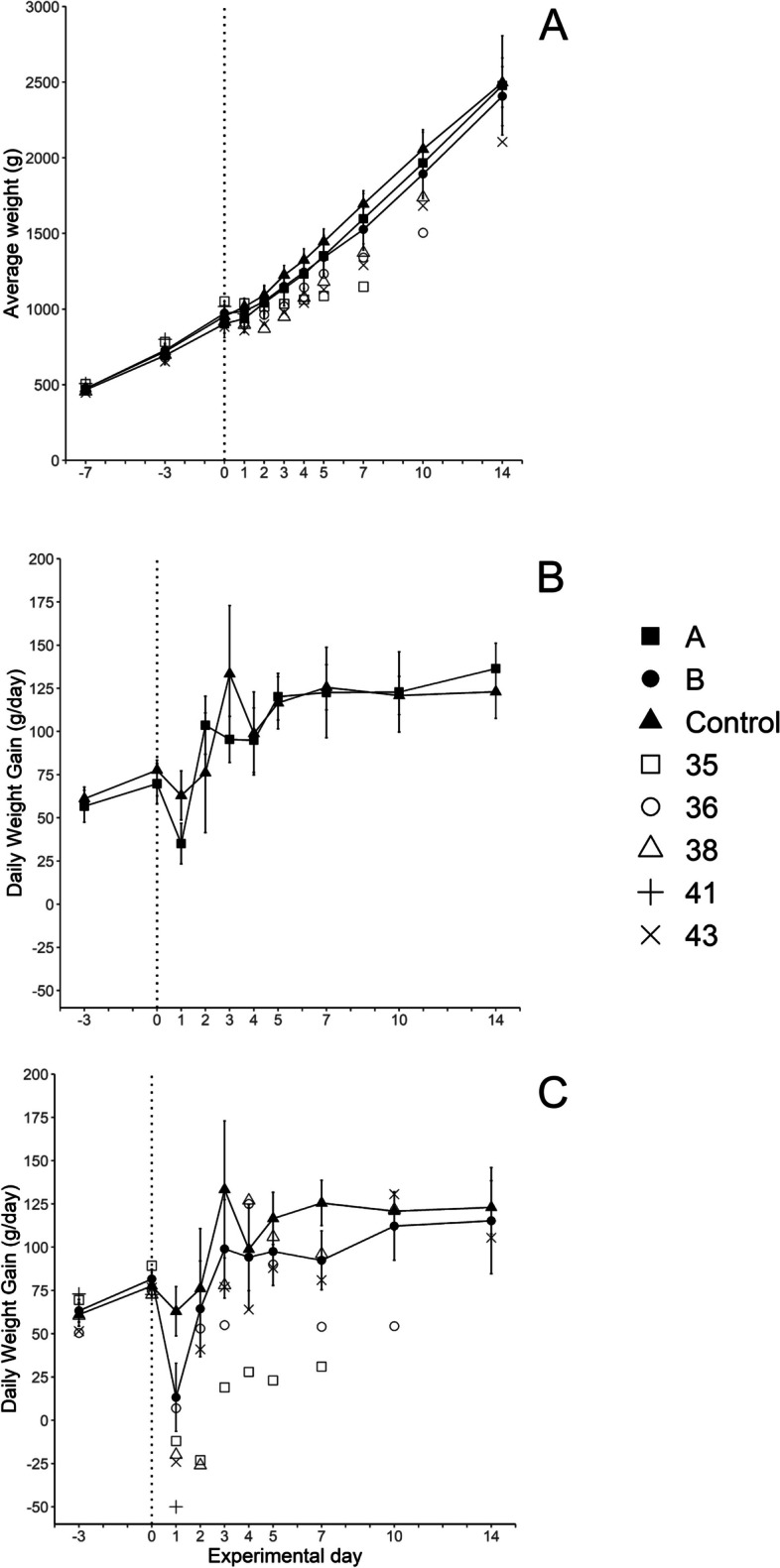
Body weight **A** and daily weight gain **B**, **C** for control chickens (filled triangles, 10 ≥ *n* ≤ 15), and chickens inoculated subcutaneously with *E. coli* strain ECA18 (group A; filled squares, 10 ≥ *n* ≤ 15) or strain ECB11 (group B; filled circles, 8 ≥ *n* ≤ 15) on experimental day 0 (dotted line). Chickens were weighed on the indicated experimental days and daily weight gains were calculated for time periods between weight recordings. Body weight on day 14 PI was only measured for 10 of 15 chickens in group A, 8 of 13 in group B and 10 of 15 in the control group. Results are shown as group mean values ± 95% CI, where non-overlapping CI indicate statistically significant differences, and as individual values for chickens no 35, 36, 38, 41 and 43, respectively, from group B that had bacteraemia on one or more sampling occasions post-inoculation

The most common findings after inoculation were mild erythema, swelling, and/or blue-grey discoloration of the skin at the inoculation site in 15/15 birds in group A and 14/15 birds in group B. One chicken from each inoculation group displayed open beak breathing and reluctance to stand on day 1 and 13 PI, respectively, and were euthanized. Five chickens (one in group A and four in group B) showed ruffled feathers or mild weakness during 1–3 days PI, and one of these chickens from group B died on day 7 PI. The mortality rates (dead and euthanized birds) were 1/15 (7%) in group A, 2/15 (13%) in group B and 0/15 in the control group. Results from individual birds are summarised in Additional file S4. Prior to inoculation, there was no significant difference in mean bodyweight between the three experimental groups and the daily weight (DWG) gain was similar and uniform (Fig. 1). On day 1 PI, the weight gain was significantly reduced in chickens from groups A and B, compared to the control group (Figs. 1B and C). Some chickens with bacteraemia (see below) showed lower body weight compared to group mean values, as well as low DWG on days 1 and 2 PI (Fig. 1A and C). The chickens in group B had significantly lower body weight gain on day 7 PI, compared to the control group. One of the bacteraemic chickens showed decreased weight gain from day 0 until euthanasia (Fig. 1C).

### Post-mortem findings

Among the necropsied birds where sex was recorded (*n* = 43), 45% were females (A: 5 birds, 36%; B: 8 birds, 57%; control group: 6 birds, 43%) and 55% males (A: 9 birds, 64%; B: 6 birds, 43%; control group: 8 birds, 57%). By mistake, sex was not recorded for one chicken/group (*n* = 3). Seven (47%) of the chickens in group A, compared to 13 (87%) in group B, were diagnosed with cellulitis (*p* < 0.05, Table 2, Additional file S4), with no significant relationship to sex (*p* > 0.05).

**Table 2:** Gross findings at the injection site of *E. coli* inoculated and sham inoculated broiler chickens. *Post-mortem* examinations were performed on day 1 PI (*n* = 1), day 7 PI (*n* = 1) and days 12–14 PI (*n* = 43)

Group/ Gross finding: chickens/group (%)	Control (*n* = 15)	A (*n* = 15)	B (*n* = 15)
Discoloured skin	0 (0%)	0 (0%)	7 (47%)
Thickened skin	0 (0%)	1 (7%)	6 (40%)
Subcutaneous oedema/congestion	0 (0%)	1 (7%)	8 (53%)
Plaque-like fibrinonecrotic material	0 (0%)	7 (47%)	12 (80%)
Plaque-like lesion size (mm, range)	-	5–20	5–50

**Fig. 2:**
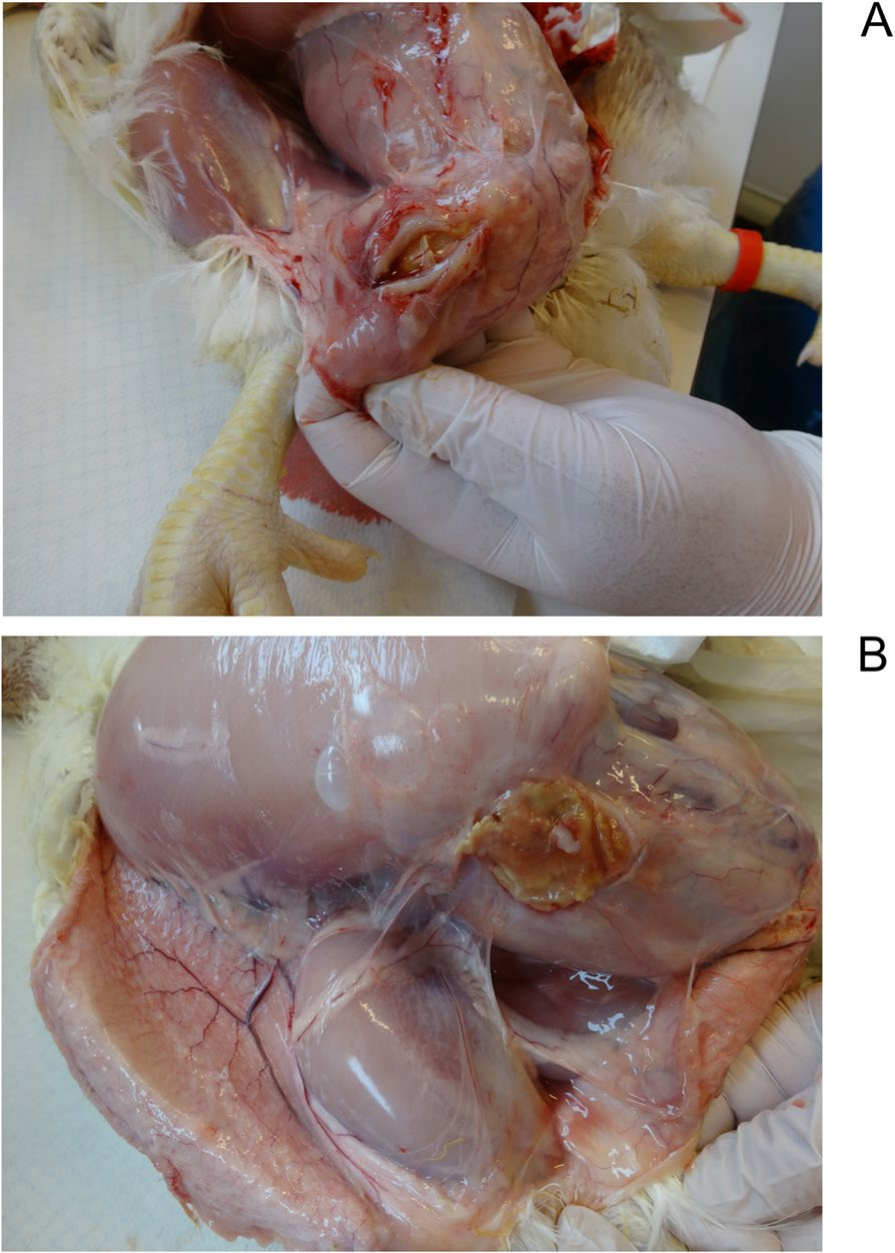
Chickens with subcutaneous lesion on the right side of the abdomen two weeks after inoculation with *E. coli* strain ECB11. **A** 40 × 10 mm incised lesion of plaque-like material enclosed in subcutaneous tissue; **B** 30 × 40 mm lesion of plaque-like material partly embedded in the abdominal wall and extending to the distal part of the pectoral muscle

The subcutaneous lesions were located at the inoculation site, and in two chickens from group B, the lesion had extended to the left side of the abdomen or to the breast. In six (86%) and 11 (85%) of the chickens diagnosed with cellulitis in group A (*n* = 7) and B (*n* = 13), respectively, plaque-like material partially or completely enclosed either in subcutaneous tissue or within the abdominal wall was observed, interpreted as a later stage of cellulitis (Fig. 2, Table 2). The chicken from group B that died on day 7 presented with a cellulitis lesion with the gross appearance similar to those found in slaughterhouses. No gross lesions suggesting bacterial infection beyond the subcutaneous tissue were observed in any of the inoculated birds. None of the chickens in the control group were diagnosed with subcutaneous lesions. Mild focal gizzard erosions were found in the majority of chickens in all experimental groups including the control birds (35/45 chickens; 73–87% per group). Samples from gizzard and caecal tonsils were negative by PCR for FAdV.

### Bacterial cultures

**Fig. 3:**
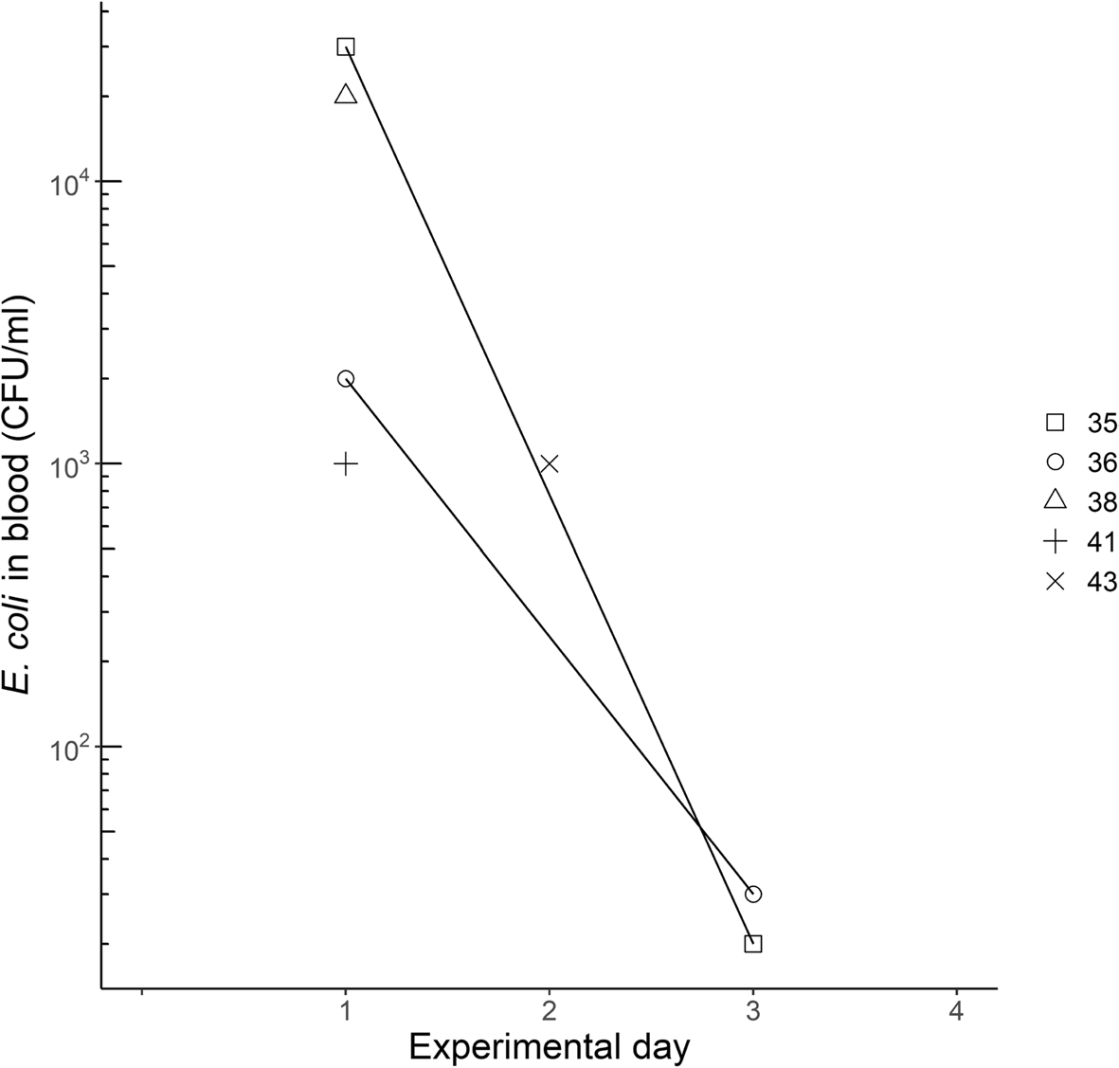
Quantification of *E. coli* by direct culture of blood collected on the indicated days after inoculation from chickens inoculated subcutaneously with *E. coli* on day 0. Results are shown as individual values for bacteraemic chickens in group B inoculated with strain ECB11 (*n* = 5)

Prior to *E. coli* inoculation (day −4), all blood cultures were negative. On days 1–3 PI, *E. coli* was isolated from blood from five chickens in group B (Fig. 3, Additional file S4). None of the chickens in group A or the control group were culture-positive in blood. At *post-mortem* examination, one of the two chickens in group B that died or were euthanized on day 1–7, had pure culture of *E. coli* in all three swab samples (the subcutaneous tissue of the inoculation site, the pericardium and the spleen parenchyma). The other chicken had pure culture of *E. coli* in the sample from the cellulitis lesion. At the end of the experiment, one chicken in group A and three chickens in group B had *E. coli* in pure culture in samples from the inoculation site (Additional file S4). The remaining samples for all groups showed either no growth (predominantly samples from the pericardium and spleen parenchyma) or sparse growth of mixed flora, with or without colonies of *E. coli* (predominantly from the inoculation site).

### Phenotypic antimicrobial susceptibility

With the exception of strain ECB11 that showed phenotypic resistance to gentamicin, the two inoculated strains were susceptible to all tested antimicrobials. Enrofloxacin and neomycin could not be assessed as there were no MIC breakpoints available [21].

### Genomic characteristics

A summary of the genomic characteristics of the two inoculation strains ECA18 and ECB11 can be found in Table 3.

**Table 3:** Genomic characteristics of the two *E. coli* strains used for experimental inoculation

Strain	Serotype	Sequence type	Virulence genes^a^	Accession numbers
ECA18	O24:H4	117	*chuA fyuA hlyF ireA irp2 iss iucC iutA lpfA neuC ompT papC pic sitA terC traT vat*	CCUG 77077^b^ ERR12344567^c^
ECB11	O153:H9	38	*air chuA cia cvaC eilA etsC gad hlyF hra iha iroN iss kpsE kpsMII mchF ompT sitA terC traT*	CCUG 77079^b^ ERR12344575^c^

^a^The following proteins are related to the genes: *air*: enteroaggregative immunoglobulin repeat protein; *chuA*: outer membrane hemin receptor; *cia*: colicin ia; *cvaC*: microcin C; *eilA*: *Salmonella* HilA homolog; *etsC*: putative type I secretion outer membrane protein; *gad*: glutamate decarboxylase; *fyuA*: siderophore receptor; *hlyF*: hemolysin F; *hra*: heat-resistant agglutinin; *iha*: adherence protein; *ireA*: siderophore receptor; *iroN*: enterobactin siderophore receptor protein; *irp2*: high molecular weight protein 2 non-ribosomal peptide synthetase; *iss*: increased serum survival; *iucC*: aerobactin synthetase, *iutA*: ferric aerobactin receptor; *kpsE*: capsule polysaccharide export inner-membrane protein; *kpsMII*: polysialic acid transport protein, group 2 capsule; *lpfA*: long polar fimbriae; *mchF*: transporter protein; *neuC*: polysialic acid capsule biosynthesis protein; *ompT*: outer membrane protease; *papC*: outer membrane usher P fimbriae; *pic*: serine protease autotransporter; *sitA*: iron transport protein; *terC*: Tellurium ion resistance protein; *traT*: outer membrane protein complement resistance; *vat*: vacuolating autotransporter toxin

^b^Culture Collection University of Gothenburg

^c^European Nucleotide Archive

The two subcultures of strain ECB11 that diverged phenotypically, *i.e.* light grey ECB11a or darker grey colonies ECB11b, were found to have an inverted region of 2610 bp. This inverted region was part of a prophage of 40.8 Kb consisting of 45 phage genes, most similar to the Mu-like *Burkholderia cenocepacia* phage BcepMu (69.7% identity, 25% coverage, accession number NC_005882.1; [44]). The analysed *E. coli* isolates from blood (*n* = 3, group B) and subcutaneous tissue (*n* = 2, one isolate from group A and B, respectively) were identical to the inocula in terms of serotype, sequence type and virulence genes. One SNP was detected in the isolate from subcutaneous tissue from group B and in one isolate from blood. The SNPs were located in the *yhhQ* gene (isolate from subcutaneous tissue; protein related to gene: queuosine precursor transporter) and *ygeH* gene (isolate from blood; protein related to gene: HilA family transcriptional regulator).

### Blood leukocyte counts

**Fig. 4:**
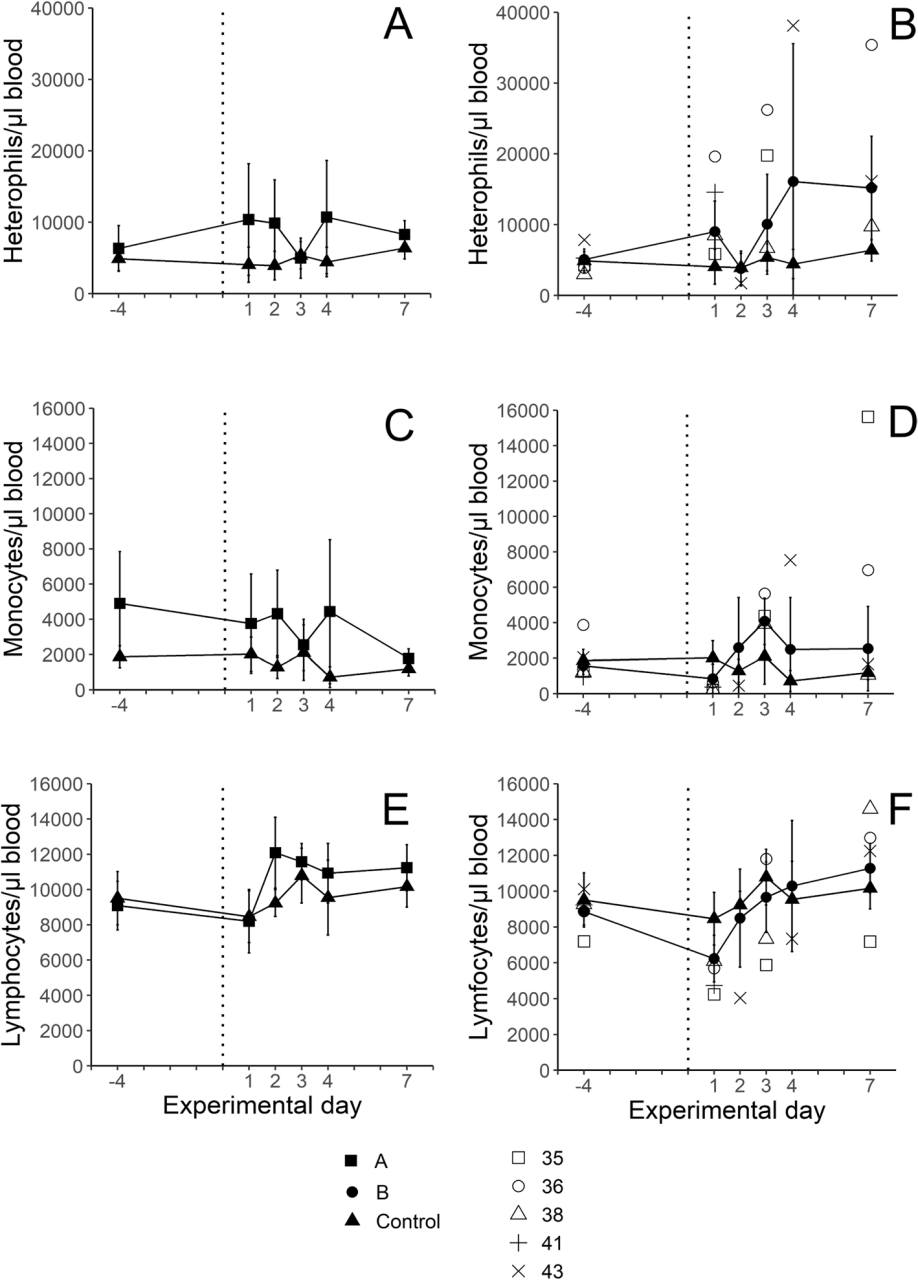
Numbers of heterophils **A**, **B** monocytes **C**, **D** and lymphocytes **E**, **F** in blood collected on the indicated days from control chickens (filled triangles, 7 ≥ *n* ≤ 15) and chickens inoculated subcutaneously with *E. coli* strain ECA18 (group A; filled squares, 7 ≥ *n* ≤ 15) or strain ECB11 (group B; filled circles, 6 ≥ *n* ≤ 15) on experimental day 0 (dotted line). Results are shown as group mean values ± 95% CI, where non-overlapping CI indicate statistically significant differences, and as individual values for chickens no 35, 36, 38, 41 and 43, respectively, from group B that had bacteraemia on one or more sampling occasions post-inoculation. For details, see the materials and methods section

Absolute counts of circulating heterophils, monocytes, thrombocytes, lymphocytes and lymphocyte subpopulations in blood collected on day −4 before inoculation and days 1–4, and 7 PI are shown in Fig. 4 and Additional file S5–S7. The heterophil numbers varied more PI in *E. coli* inoculated birds, compared to the control birds (Fig. 4A and B). Three of the bacteraemic chickens in group B showed considerably elevated heterophil numbers and the mean heterophil number in group B was three-fold higher on days 4 and 7 PI, compared to the control group (Fig. 4B). No differences were observed for monocytes (Fig. 4C and D) and thrombocytes (Additional file S5). Chickens in group B showed decreased numbers of lymphocytes on day 1 PI, although not significant, compared to the control chickens, while some of the birds with bacteraemia showed a more pronounced decrease (Fig. 4E and F). Among the different lymphocyte subpopulations, the numbers of TCRγδ+CD8αβ+ cells were significantly decreased in blood from chickens in group B on day 1 PI, compared to the control group (Additional file S7L). The numbers of B-cells (Additional file S6) as well as CD4+CD8α + , CD4+CD8-, CD4-CD8𝛼β+ , CD4-CD8𝛼𝛼+ , TCR𝛾/𝛿+CD8𝛼𝛼 + and TCR𝛾/𝛿+CD8- cells (Additional file S7), tended to be decreased in blood from chickens in group B on day 1, and all of the bacteraemic chickens also showed low numbers PI of at least one of these cells types. Among the T-cell populations, the proportion of cells expressing CD25 in the CD4+CD8-, CD4+ CD8𝛼+ , CD4-CD8𝛼β+ and TCR𝛾/𝛿+CD8- populations (Additional file S8) were approximately 8, 2, 0.4 and 8% respectively, and did not differ between the inoculated groups and the control group.

### Expression of MRC1L‑B and CD45

**Fig. 5:**
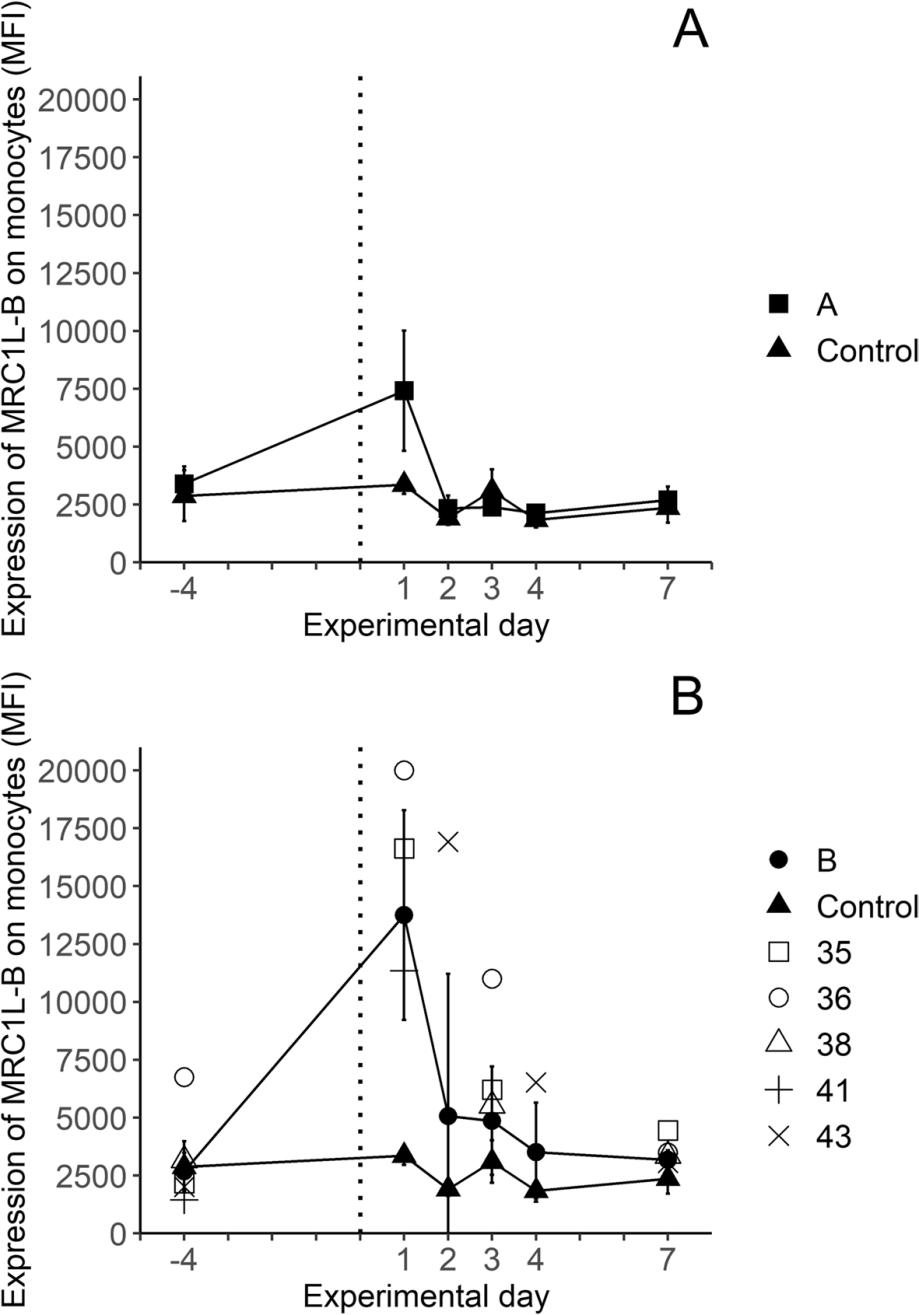
Expression of MRC1L-B (**A**, **B**) on monocytes in blood collected on the indicated days from control chickens (filled triangles, 7 ≥ *n* ≤ 15) and chickens inoculated subcutaneously with *E. coli* strain ECA18 (group A; filled squares, 7 ≥ *n* ≤ 15) or strain ECB11 (group B; filled circles, 6 ≥ *n* ≤ 15) on experimental day 0 (dotted line). Results are shown as group mean values ± 95% CI, where non-overlapping CI indicate statistically significant differences, and as individual values for chickens no 35, 36, 38, 41 and 43, respectively, from group B that had bacteraemia on one or more sampling occasions post-inoculation

The levels of cell-surface expression of the mannose receptor MRC1L-B on monocytes, and of CD45 on monocytes, heterophils, lymphocytes and thrombocytes, were monitored as mean fluorescence intensity (MFI) in their respective gate (Additional file S1). Results showed that the expression of MRC1L-B was significantly increased on monocytes from chickens in both groups A and B on day 1 PI, compared to control chickens (Fig. 5). This increase was transient and for chickens in group A, the MRC1L-B expression had returned to pre-infection levels on day 2 PI while for chickens in group B, it gradually returned to pre-infection levels until day 4.

Expression of CD45 on monocytes and heterophils varied between groups already before inoculation, and for all analysed leukocyte populations, a large variation in CD45 expression between the sampling occasions was observed (Additional file S9). Significant changes in the CD45 expression were seen in both *E. coli* inoculated groups, compared to the control group. On days 2, 3 and 7 PI, the expression was lower on monocytes (Additional file S9C and D) and the expression on heterophils was lower in group A on days 2 and 7 PI (Additional file S9A and B). Further, the expression of CD45 on lymphocytes was lower on days 1 and 2 PI in group A, and in group B on day 2 (Additional file S9E and F).

### *E. coli* leukocyte adherence

**Fig. 6:**
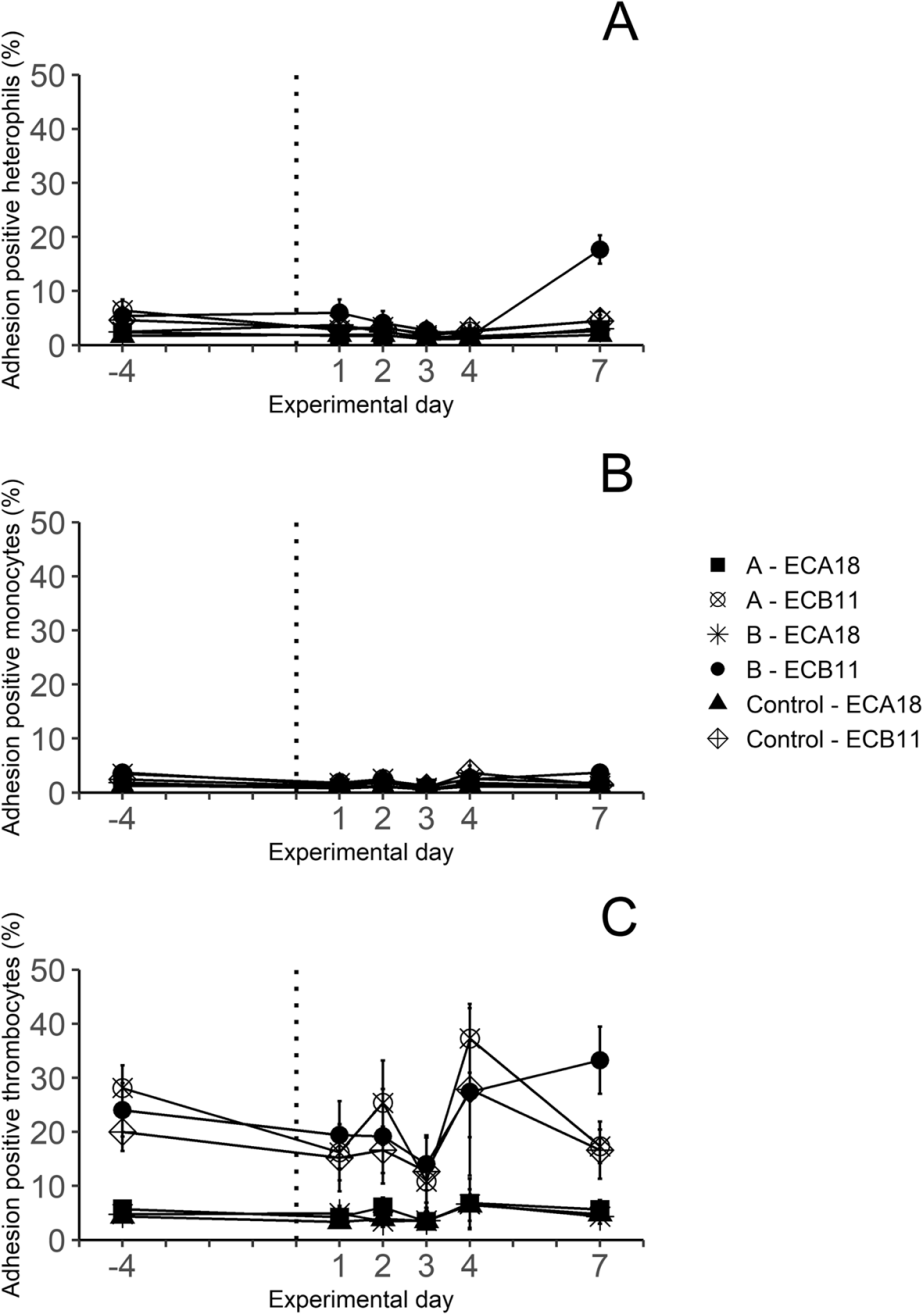
Adhesion of *E. coli* to different leukocyte populations: (**a**) heterophils, (**b**) monocytes and (**c**) thrombocytes in whole blood cultures established with blood collected on the indicated days from control chickens (7 ≥ *n* ≤ 15) and chickens inoculated subcutaneously with *E. coli* strain ECA18 (group A, 7 ≥ *n* ≤ 15) or strain ECB11 (group B, 6 ≥ *n* ≤ 15) on experimental day 0 (dotted line). Cultures were incubated for 15 min with either strain ECA18 (A) or strain ECB11 (B), for details see materials and methods. Results are shown as group mean values ± 95% CI, where non-overlapping CI indicate statistically significant differences

In cultures set up prior to *E. coli* inoculation and with blood from the control birds throughout the experiment, up to 5% of heterophils and monocytes and approximately 25% of thrombocytes adhered to strain ECB11 (Fig. 6A–C). The proportions of heterophils and monocytes adhering to strain ECA18 tended generally to be lower. Approximately 5% of thrombocytes adhered to ECA18, and the significant difference between adherence of thrombocytes to ECA18 or ECB11 persisted throughout the experiment (Fig. 6C). On day 7 PI, in cultures set up from chickens in group B, all three cell types showed a significant increase of the proportions of cells adhering to *E. coli* strain ECB11, compared to in cultures from controls. The proportion of heterophils showed an approximate fourfold increase (Fig. 6A–C) while monocytes and thrombocytes showed a low numerical increase. No differences in the proportions of adhered leukocytes to strain ECA18 were observed in cultures set up from chickens in group B. In cultures set up from chickens in group A, no differences were observed for either strain ECA11 or ECA18. Correlations within chicken between the proportions of cells that adhered to *E. coli* and the IgY titers to the respective *E. coli* strain, were analysed for the results obtained on day −4 and 7. In chickens from group B, the proportions of heterophils that adhered to *E. coli* strain ECB11 was positively correlated to IgY titers to ECB11 (r_s_ = 0.70, *p* < 0.05), while no such correlations were found for monocytes (r_s_ = 0.24, *p* > 0.05) or thrombocytes (r_s_ = 0.24, *p* > 0.05). In group A, there was a weak to intermediate negative correlation between the proportions of monocytes that adhered to strain ECB11 and titers to ECB11 (r_s_ = −0.42, *p* < 0.05), while no such correlations were found for heterophils (r_s_ = −0.11, *p* > 0.05) or thrombocytes (r_s_ = −0.18, *p* > 0.05). Further, the proportions of monocytes that adhered to strain ECA18 in group A, showed a weak to intermediate negative correlation to IgY titers to strain ECA18 (r_s_ = −0.46, *p* < 0.05), while no such correlation were found for heterophils (r_s_ = 0.27. *p* > 0.05) or thrombocytes (r_s_ = 0.11, *p* > 0.05). In group B, there was no correlation between the proportions of cells that adhered to strain ECA18 and titers to ECA18 for any cell type (heterophils: r_s_ = 0.14, *p* > 0.05; monocytes: r_s_ = −0.33,* p* > 0.05 and thrombocytes: r_s_ = −0.16, *p* > 0.05).

### *E. coli*-specific IgY responses

**Fig. 7:**
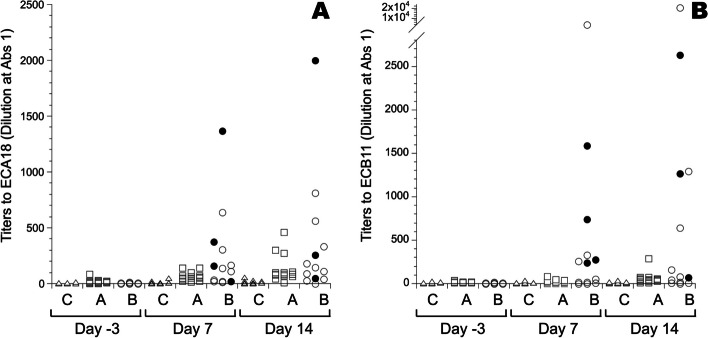
IgY titers to a sonicated antigen preparation of ECA18 **A** or ECB11 **B** in sera collected on the indicated experimental days from control chickens (C; triangles, 14 ≥ *n* ≤ 15) and chickens inoculated subcutaneously with *E. coli* strain ECA18 (group A; squares, *n* = 15) or strain ECB11 (group B; circles, 13 ≥ *n* ≤ 15) on experimental day 0. Results are shown as individual values for all chickens with titers > 1, with filled symbols for chickens from group B that had bacteraemia on one or more sampling occasions post-inoculation

Titers of IgY were low to both strains ECA18 and ECB11 before inoculation in all groups, and titers remained low in the control group after inoculation (Fig. 7). Mean titers to antigen ECA18 were significantly increased on days 7 and 14 PI for chickens in group A and B, compared to those in the control group (Additional file S10). Only a limited number of chickens showed increased titers > 200 (3 of 15 day 14 for group A; 4 of 14 day 7 and 5 of 13 day 14 for group B) to antigen from strain ECA18 (Fig. 7A). Mean titers of the antigen to strain ECB11 were significantly increased on days 7 and 14 PI for chickens in group B, and on day 14 for chickens in group A (Additional file S10), compared to those in the control group. Moreover, approximately half of the chickens (7 of 14 on day 7 and 5 of 13 on day 14) in group B showed titers > 200, while only one chicken in group A had a titer of > 200 to the antigen from strain ECB11 on day 14 (Fig. 7B). For both antigens, some of the bacteraemic chickens in group B were among those displaying titers of > 200 (Fig. 7). Not all samples were deemed positive (titer > 1) for IgY to *E. coli* (Additional file S10). There was a significant positive correlation within chicken at each sampling occasion between IgY titers to strain ECA18 or ECB11 for all groups (*p* < 0.05, r_s_ = 0.79).

## Discussion

In the present study, broiler chickens were inoculated with *E. coli* subcutaneously with the aim to induce cellulitis. Clinical signs and selected immune parameters were evaluated. The bacteria that were re-isolated from lesions and blood were shown to be identical to the inoculation strains. The main finding among the evaluated immune parameters was a rapid transient increase in MRC1L-B expression on circulating monocytes in all *E. coli* inoculated chickens.

The most likely time-point for broiler chickens to develop cellulitis during grow-out is not known. Generally, affected birds are identified at slaughter. At this stage, the lesions are characterized by a necrotic subcutaneous exudate (plaque), but signs suggestive of chronic inflammation are rarely seen [5, 14, 45]. In the present study, when chickens were euthanized two weeks after inoculation, we observed necrotic material partly embedded in the subcutaneous tissue which were interpreted as a later stage of cellulitis in the majority of birds. In two cases, more locally extensive lesions were observed. An earlier study by Peighambari et al*.* reported microscopic evidence of chronic inflammation when inoculated chickens were sampled ten days after inoculation with *E. coli* in a cellulitis experimental model [4], but as microscopy was not performed in the present study, the histopathological features could not be compared. Lesions in the current study were smaller than the plaque described in a study performed on carcasses at slaughter [14], which indicates that the current infection was mild and/or that the chickens had already cleared the bacterial infection when necropsied. However, in birds on commercial farms, *E. coli* presumably enters the tissue through skin lesions. Thus, there are most likely some differences in how cellulitis develops experimentally versus in a commercial situation that may influence the kinetics of lesion development.

As previously described, few birds in this study showed clinical signs of depression following inoculation. This observation mirrors what is reported from farms [5]. The body weight PI showed no overall difference between the A, B and control groups, while the DWG was transiently significantly lower on day 1 PI for group A and B, and on day 7 PI for group B, compared to the control group. The body weight gain can be affected by an acute inflammatory response induced by *E. coli* as reported by others [7, 46, 47]. The reduced DWG after inoculation in our study suggests that even in the absence of other clinical signs, the performance of broilers may be affected by cellulitis caused by *E*. *coli*.

A pronounced, transient increase of MRC1L-B expression on monocytes was observed for all inoculated chickens on day 1 PI regardless of *E. coli* strain, clinical signs, or detection of bacteraemia. This increase was most prominent for chickens in group B, and several of the inoculated chickens that developed bacteraemia during the experiment also showed increased MRC1L-B expression on days 3–7 PI. Interestingly, these results are in agreement with our previous observations during experimental inoculation of chickens with the Gram-positive bacterium *Erysipelothrix rhusiopathiae* [37, 39]. In those studies, there was a prompt, transient, increased expression of MRC1L-B expression on circulating monocytes and a higher or prolonged high expression correlating with pronounced clinical signs and/or prolonged bacteraemia. Thus, this response might be a consistent feature of chicken monocytes upon bacterial infection. The increased MRC1L-B expression could reflect an altered cell-surface expression following *e.g.* activation or maturation, or a re-distribution of monocyte populations with different MRC1L-B expression. Altered MRC1L-B expression has been observed upon in vitro maturation of chicken monocytes [48], and as a response to in vitro stimulation with innate defence peptides [49]. Among chicken blood monocytes, subtypes with high or low MRC1L-B expression have been identified using single cell transcriptomics and evidence of functional differences between these were inferred based on their mRNA expression profiles [50]. Moreover, in a study of chicken spleen macrophages, a phenotype with MRC1L-B^high^MHCII^low^ expression was identified, which expanded upon LPS stimulation and showed a high phagocytic capacity [51]. Hence, the observed alterations in MRC1L-B expression may reflect an innate response to promote clearance of the bacterial infection, that is quickly down-regulated when the infection is controlled.

On day 7 PI, there was a significant increase of in vitro adherence to *E. coli* strain ECB11 in cultures from chickens in group B, that was not seen to strain ECA18 or for group A. Thus, the timing and the putative strain-specificity of this observation suggest that the increased adherence was due to opsonisation by ECB11 strain-specific antibodies. In our study, the increased adherence to *E. coli* was most pronounced for heterophils. In analogy, early work on in vitro phagocytosis of *Salmonella* Enteritidis by chicken leukocytes showed that both heterophil and monocyte phagocytosis was enhanced by bacteria-specific antibodies, but depending on the assay used, the effect was often most pronounced for heterophils [52]. Moreover, we found that approximately 50% of the chickens in group B had high titers of IgY directed to strain ECB11 in samples collected on day 7 PI. Further, the titers in group B strongly correlated with adherence of *E. coli* strain ECB11 to heterophils, which supports that the observed effects in vitro were due to antibody opsonisation. However, the strong correlation between antibody titers to strains ECA18 and ECB11 suggests cross-reactivity of IgY to both strains in our ELISA-assay, which was not seen in the adherence assay. The two strains represent different serotypes, O24:H4 for ECA18 and O153:H9 for ECB11, hence strain-specific antibodies should be able to distinguish between them. However, the sonicated whole-bacteria lysate used for coating in the ELISA assay might have resulted in detection of IgY recognising some of the common and not strain-specific *E. coli* antigens. Cross reactivity of IgY to *E. coli* of different serotypes has previously been reported using sonicated whole-bacteria lysates in ELISA assays [19, 53], and is probably difficult to avoid without the use of purified, serotype-specific bacterial surface antigens. In our adherence assay, intact bacteria were used that most likely limited the binding of IgY to surface antigens and thus increased the serotype specificity of antibody opsonisation. No evidence of antibody-mediated opsonisation was observed in cultures from chickens in group A, which was likely due to the low levels of circulating IgY recognising *E. coli* in these chickens at the time points when bacterial adherence was assessed.

The birds with bacteraemia generally had a higher antibody titer at 14 days PI, as compared to the birds without bacteraemia. This suggests that a local *E. coli* infection may not be sufficient to elicit a detectable antibody response. In the present study, the infection was mainly local and the bacteria may have been cleared before mounting a substantial antibody response. This was also supported in the bacterial samples taken at *post-mortem* examination, as chickens sampled within one week after inoculation had pure culture of *E. coli*, while birds sampled day 14 PI generally had a nonspecific mixed flora with few *E. coli* colonies.

Only five birds, all inoculated with *E. coli* strain ECB11, displayed bacteraemia that persisted up to three days after inoculation. In an earlier study by Gomis et al*.*, nine out of ten chickens inoculated subcutaneously with *E. coli* displayed bacteraemia one day after infection, and bacteraemia could be detected in some birds on day 7 [7]. Compared to the present study, the levels of mortality and systemic spread of *E. coli* in that study was also higher, which may speculatively be associated with a higher infection dose of *E. coli* (up to 10^9^ CFU/mL) and/or a more virulent *E. coli* strain [7]. The *E. coli* strains used for inoculation in the present study represented sequence types and carried virulence genes that are commonly found in APEC. It should however be noted that there is no clear definition of the APEC pathotype, although there are some combinations of virulence genes that have been used to distinguish APEC from commensal *E. coli* [54, 55]. Further, the results indicates that strain ECB11 (group B) was more virulent than strain ECA18 (group A), with respect to bacteraemia, frequency and gross findings of cellulitis, and the level of immune responses. This could have been due to the fact that group B received a 1.6 times higher inoculation dose than group A, but also a result of the strain characteristics.

The chickens in the present study did not show any major alterations in the blood leukocyte counts, which is in line with the finding of a localised and mild outcome. Some of the bacteraemic chickens in group B showed increased numbers of heterophils and monocytes in the circulation. These cell types are typically associated with the first line defence against bacterial infections [56] and may be seen in bacteraemic chickens and/or chickens with more severe clinical signs. Further, chickens inoculated with strain ECB11 tended to have transiently decreased numbers of circulating lymphocytes, in total as well as in the subpopulations studied, early (day 1) PI. This decrease was statistically significant for TCRγδ +CD8αβ+ cells. In addition, two of the chickens with bacteraemia showed lower numbers of all lymphocyte populations for a prolonged period after *E. coli* inoculation, similar to what was observed after *E. rhusiopathiae* infection in chickens [37, 39]. This observation may hypothetically be an effect of bacterial toxins and/or reflect infection-induced lymphocyte retention. However, the virulence genes found exclusively in strain ECB11 in the present study, have not been associated with such toxin that specifically affects lymphocytes.

The cause of the gizzard erosion and ulceration (GEU) observed in this study was not determined. FAdV species A can cause severe GEU in young chickens, but it was not detected by PCR. Other possible causes include *e.g.* mycotoxins and biogenic amines [57], but these were not further investigated as it was beyond the scope of our study. As the GEU in this study were mild and observed in the majority of chickens including control birds, any effects on *e.g.* blood leukocyte counts should have affected all groups.

## Conclusions

In the present study, the experimental model used in broiler chickens was effective to induce a localized subcutaneous lesion with the presence of plaque-like material at the inoculation site in the majority of birds. Some of the lesions were partly embedded in subcutaneous tissue and were interpreted as a later stage of cellulitis, which indicates that cellulitis in commercial broilers develops during the final stage of the rearing period. Moreover, the infection elicited a rapid activation or redistribution of monocytes into a profile that may enhance elimination of bacteria. Immune responses were more prominent when inoculating an *E. coli* strain with a presumed higher virulence. Hence, this study showed some novel findings on the chicken innate immune response to *E. coli* infection. Future studies should focus on improved understanding of the host–pathogen interaction involved in cellulitis, in terms of animal husbandry, immune response, host genetics and *E. coli* virulence traits.

## Supplementary Information

Additional file 1. Gating strategy for of heterophils, monocytes, lymphocytes, thrombocytes and B-cells in whole blood. Gating strategy to define different leukocyte populations for flow cytometric analysis of leukocyte populations in whole blood using panel 1 (Table 1). Identification of heterophils, monocytes, lymphocytes, thrombocytes, B-cells and counting beads through singlet gating, FSC/SSC characteristics and using CD45-PerCp/Cy5.5, CD41/61-Fitc, KUL01-PE (MRC1L-B) and Bu-1-pacific blue. From the initial dot-plot in A, beads were identified as high Fitc and high PE in B. From A singlet gating (FSC-H vs FSC-A) was performed in C. From the singlet gate in C, CD45 high and CD45 intermediate/SSC low events were gated in D. From the gate in D, the SSC high events were gated in E and from this gate CD45 high/PE low events were gated in F and back-gated as heterophils (high SSC) in G. From the gate in D, thrombocytes (CD41/61 positive) and KUL01 positive events were gated in H. From the KUL01 gate in H, events were gated as monocytes based on FSC/SSC characteristics in I. Non-thrombocyte events in H were gated on FSC low/SSC low profile as lymphocytes in J. From the CD45 gate in D Bu-1 positive events were gated in K and the Bu-1 positive events were gated as B-cells based on FSC/SSC characteristics in L. A representative chicken blood sample from a B group chicken (no. 37) on day 1 is shown

Additional file 2. Gating strategy for TCR𝛾/𝛿+ , CD8+ and CD4+ lymphocyte subpopulations in whole blood. Gating strategy for flow cytometric analysis of leukocyte populations in whole blood using panel 2 (Table 1 ). Identification of lymphocyte sub populations TCR𝛾/𝛿+CD8-, TCR𝛾/𝛿+CD8𝛼β + , TCR𝛾/𝛿+CD8𝛼𝛼 + , TCR𝛾/𝛿-CD8𝛼β + (CTL), TCR𝛾/𝛿-CD8𝛼𝛼+ , CD4+CD8-, CD4+CD8𝛼𝛼+ and CD25 expression on some of these as well as counting beads through singlet gating, FSC/SSC characteristics and using CD41/61-Fitc, CD8β-PE, CD8𝛼-Cy5, CD4-pacific blue, TCR1-PerCPCy5.5 and CD25-PECy5. From the initial dot-plot in A, beads were identified as high Fitc and high PE in B. From A singlet gating (FSC-H vs FSC-A) was performed in C. From the singlet gate in C non-thrombocyte events (CD41/61 negative) were identified in D and were gated on a FSC low/SSC low profile as lymphoid in E. Lymphoid events from E were gated as TCR𝛾/𝛿+ in F. TCR𝛾/𝛿+ events were defined according to CD8 expression as TCR𝛾/𝛿+CD8-, TCR𝛾/𝛿+CD8𝛼β + or TCR𝛾/𝛿+CD8𝛼𝛼+ in G and CD25 expression on TCR𝛾/𝛿+CD8- in H. Non-TCR𝛾/𝛿+ events from F were defined according to CD4 expression in I and CD4 positive events defined according to CD8 expression as CD4+CD8- or CD4+CD8𝛼𝛼+ in J and CD25 expression on these populations were defined in K and L. CD4 negative events defined in I were defined according to CD8 expression in M as TCR𝛾/𝛿-CD8𝛼β+ (CTL) or TCR𝛾/𝛿-CD8𝛼𝛼+ and CD25 expression on TCR𝛾/𝛿-CD8𝛼β+ was defined N. The CD4-CD8𝛼𝛼+ , TCR𝛾/𝛿+CD8𝛼𝛼+ and TCR𝛾/𝛿+CD8𝛼β+ populations had very few events (< 77 in mean events) and CD25 expression was therefore not relevant to analyse for these populations. A representative chicken blood sample from a group B chicken (no. 38) on day 1 is shown

Additional file 3. Gating strategy for *E. coli* adherence to leukocyte populations. Gating strategy for flow cytometric analysis of *E. coli * adhered to heterophils, monocytes and thrombocytes, respectively, after incubation in vitro in whole blood cultures using panel 3 (Table 1). Identification of *E. coli* adhered to heterophils, monocytes, and thrombocytes, through singlet gating, FSC/SSC characteristics and using CD45-PerCp/Cy5.5, CD41/61-Fitc, KUL01-PE (MRC1L-B) and FarRed labelled * E. coli* . From the initial dot-plot in A singlet gating (FSC-H vs FSC-A) was performed in B. From the singlet gate in B CD45 high and CD45 intermediate/SSC low events were gated in C. From the gate in C the SSC high events were gated in D and from this gate CD45 high/PE low events were gated in E and back-gated as heterophils (high SSC) in F. Heterophils positive for adhered * E. coli* were defined in G, in G1 a control culture without bacteria is shown and in G2 a culture incubated with* E. coli* is shown. From the gate in C, thrombocytes (CD41/61 positive) and KUL01 positive events were gated in H. Thrombocytes positive for adhered *E. coli* were defined in I, in I1 a control culture without bacteria is shown and in I2 a culture incubated with *E. coli* is shown. From the KUL01 gate in H events were gated as monocytes based on FSC/SSC characteristics in J. Monocytes positive for adhered *E. coli* were defined in K, in K1 a control culture without bacteria is shown and in K2 a culture incubated with * E. coli* is shown. A representative whole blood culture from a chicken from group B (no. 38) on day 7 is shown with FarRed labelled *E. coli* of strain ECB11

Additional file 4. Summary of findings. Summary of individual general appearance, culture results, outcome and *post-mortem* findings in control chickens (*n*=15) and chickens inoculated subcutaneously on experimental day 0 with *E. coli* strain ECA18 (group A, *n*=15) or strain ECB11 (group B, *n*=15). Only chickens with findings are included

Additional file 5. Numbers of thrombocytes. Numbers of thrombocytes in blood collected on the indicated days from control chickens (filled triangles, 7≥*n*≤15) and chickens inoculated subcutaneously with *E. coli* strain ECA18 (group A; filled squares, 7≥n≤15) or strain ECB11 (group B; filled circles, 6≥*n*≤15) on experimental day 0 (dotted line). Results are shown as group mean values ± 95% CI, where non-overlapping CI indicate significant differences, and as individual values for chickens no 35, 36, 38, 41 and 43, respectively, from group B that had bacteraemia on one or more sampling occasions post-inoculation

Additional file 6. Numbers of B-cells. Numbers of B-cells in blood collected on the indicated days from control chickens (filled triangles, 7≥*n*≤15) and chickens inoculated subcutaneously with *E. coli* strain ECA18 (group A; filled squares, 7≥*n*≤15) or strain ECB11 (group B; filled circles, 6≥*n*≤15) on experimental day 0 (dotted line). Results are shown as group mean values ± 95% CI, where non-overlapping CI indicate statistically significant differences, and as individual values for chickens no 35, 36, 38, 41 and 43, respectively, from group B that had bacteraemia on one or more sampling occasions post-inoculation

Additional file 7. Numbers of TCRγ/δ+, CD8+ and CD4+ lymphocyte subpopulations. Numbers of CD4+CD8αα+ cells (A, B), CD4+CD8- cells (C, D); CD4-CD8αα+ cells (E, F), CD4-CD8αβ+ cells (G, H), TCRγ/δ+CD8αα+ cells (I, J), TCRγ/δ+CD8αβ+ cells (K, L) and TCRγ/δ+CD8- cells (M, N); in blood collected on the indicated days from control chickens (filled triangles, 7≥*n*≤15) and chickens inoculated subcutaneously with *E. coli *strain ECA18 (group A; filled squares, 7≥*n*≤15) or strain ECB11 (group B; filled circles, 6≥*n*≤15) on experimental day 0 (dotted line). Results are shown as group mean values ± 95% CI, where non-overlapping CI indicate statistically significant differences, and as individual values no 35, 36, 38, 41 and 43, respectively, for chickens from group B that had bacteraemia on one or more sampling occasions post-inoculation

Additional file 8. Proportions of CD25+ cells in TCRγ/δ+, CD8+ and CD4+ lymphocyte subpopulations. Proportions of CD25+ cells in the CD4+CD8- (A, B), CD4+CD8α+ (C, D), CD4-CD8αβ+ (E, F) and TCRγδ+CD8- (G, H) populations in blood collected on the indicated days from control chickens (filled triangles, 7≥*n*≤15) and chickens inoculated subcutaneously with* E. coli *strain ECA18 (group A; filled squares, 7≥*n*≤15) or strain ECB11 (group B; filled circles, 6≥*n*≤15) on experimental day 0 (dotted line). Results are shown as group mean values ± 95% CI, where non-overlapping CI indicate statistically significant differences, and as individual values for chickens no 35, 36, 38, 41 and 43, respectively, from group B that had bacteraemia on one or more sampling occasions post-inoculation

Additional file 9. Cell surface expression of CD45. Expression of CD45 on heterophils (A, B), monocytes (C, D), lymphocytes (E, F) and thrombocytes (G, H) in blood collected on the indicated days from control chickens (filled triangles, 7≥*n*≤15) and chickens inoculated subcutaneously with *E. coli* strain ECA18 (group A; filled squares, 7≥*n*≤15) or strain ECB11 (group B; filled circles, 6≥*n*≤15) on experimental day 0 (dotted line). Results are shown as group mean values ± 95% CI, where non-overlapping CI indicate statistically significant differences, and as individual values for chickens no 35, 36, 38, 41 and 43, respectively, from group B that had bacteraemia on one or more sampling occasions post-inoculation

Additional file 10. *E. coli* specific IgY titers. Geometric mean values and 95% CI for IgY titers to a sonicated antigen preparation of ECA18 or ECB11 in sera collected on the indicated experimental days from control chickens (C, 14≥*n*≤15) and chickens inoculated subcutaneously with *E. coli* strain ECA18 (group A, *n*=15) or strain ECB11 (group B, 13≥*n*≤15) on experimental day 0

## Data Availability

All data generated and analysed in this study are included in the article text, tables, figures and additional files. The raw datasets used and/or analysed during the current study are available from the corresponding author on reasonable request. The strains used for the experimental inoculation, ECA18 and ECB11, have been deposited at the Culture Collection University of Gothenburg in Sweden (https://www.ccug.se) with numbers CCUG 77077 (ECA18) and CCUG 77079 (ECB11). The raw sequencing data have been submitted to the European Nucleotide Archive with accession numbers ERR12344567 and ERR12344575.

## Abbreviations

APEC: Avian pathogenic *Escherichia coli*
BHI: Brain heart infusion broth
CI: Confidence interval
DWG: Daily weight gain
FAdV: Fowl adenovirus
GEU: Gizzard erosion and ulceration
MALDI-TOF MS: Matrix-assisted laser desorption ionization-time of flight mass spectrometry
MFI: Mean fluorescence intensity
MIC: Minimum inhibitory concentration
MRC1L-B: Mannose receptor C-type 1-like B
PI: Post inoculation
SNP: Single nucleotide polymorphism
