# Supplementary figures and images for: Subcutaneous inoculation of Escherichia coli in broiler chickens causes cellulitis and elicits innate and specific immune responses
Source: BMC Vet Res. 2024 Dec 2;20:545. doi: 10.1186/s12917-024-04392-2 (PMC11610265; doi:10.1186/s12917-024-04392-2)

# S1

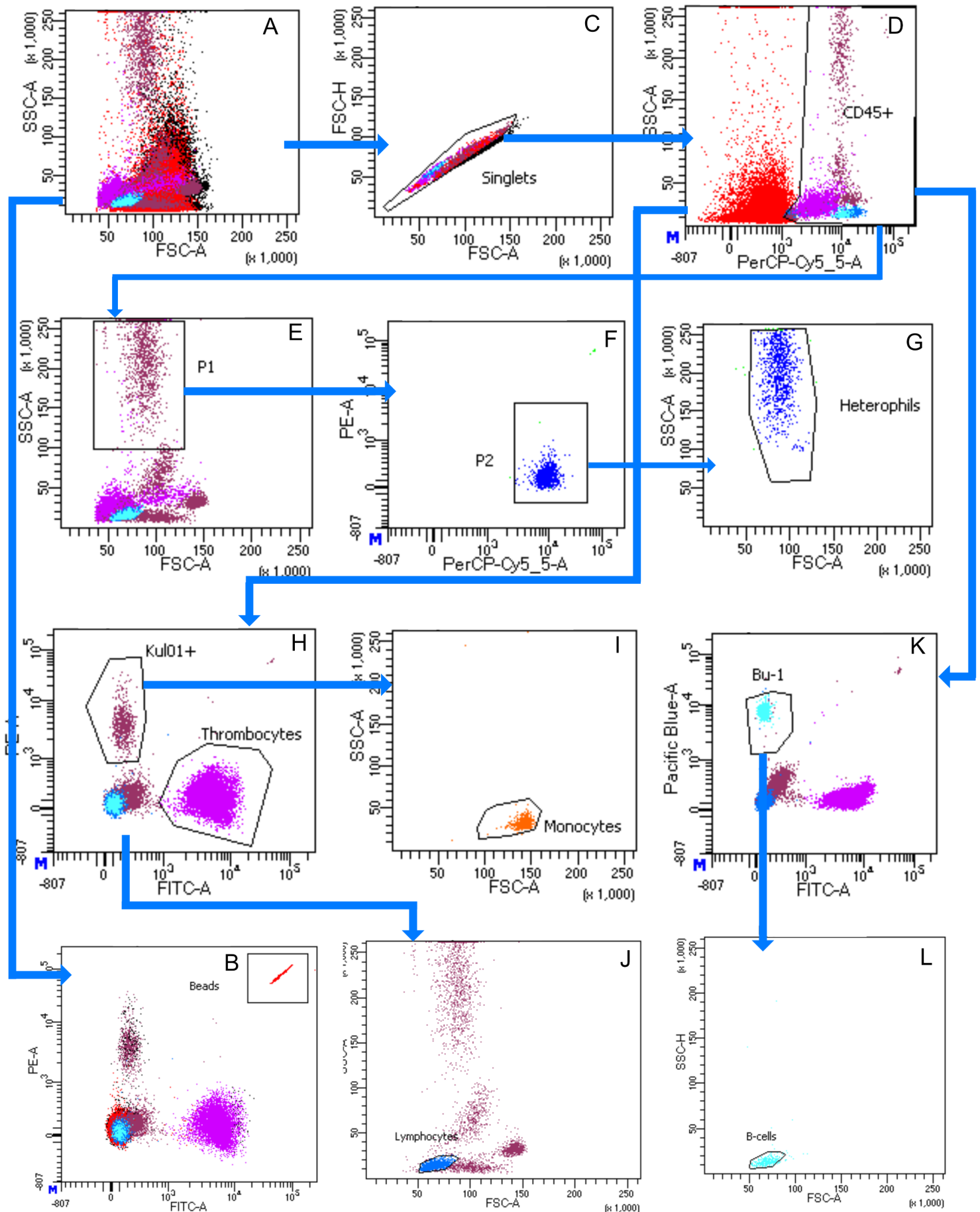

Supplement: Supplementary file 1 — Additional file 1. Gating strategy for of heterophils, monocytes, lymphocytes, thrombocytes and B-cells in whole blood. Gating strategy to define different leukocyte populations for flow cytometric analysis of leukocyte populations in whole blood using panel 1 (Table 1). Identification of heterophils, monocytes, lymphocytes, thrombocytes, B-cells and counting beads through singlet gating, FSC/SSC characteristics and using CD45-PerCp/Cy5.5, CD41/61-Fitc, KUL01-PE (MRC1L-B) and Bu-1-pacific blue. From the initial dot-plot in A, beads were identified as high Fitc and high PE in B. From A singlet gating (FSC-H vs FSC-A) was performed in C. From the singlet gate in C, CD45 high and CD45 intermediate/SSC low events were gated in D. From the gate in D, the SSC high events were gated in E and from this gate CD45 high/PE low events were gated in F and back-gated as heterophils (high SSC) in G. From the gate in D, thrombocytes (CD41/61 positive) and KUL01 positive events were gated in H. From the KUL01 gate in H, events were gated as monocytes based on FSC/SSC characteristics in I. Non-thrombocyte events in H were gated on FSC low/SSC low profile as lymphocytes in J. From the CD45 gate in D Bu-1 positive events were gated in K and the Bu-1 positive events were gated as B-cells based on FSC/SSC characteristics in L. A representative chicken blood sample from a B group chicken (no. 37) on day 1 is shown [file 12917_2024_4392_MOESM1_ESM.pdf]

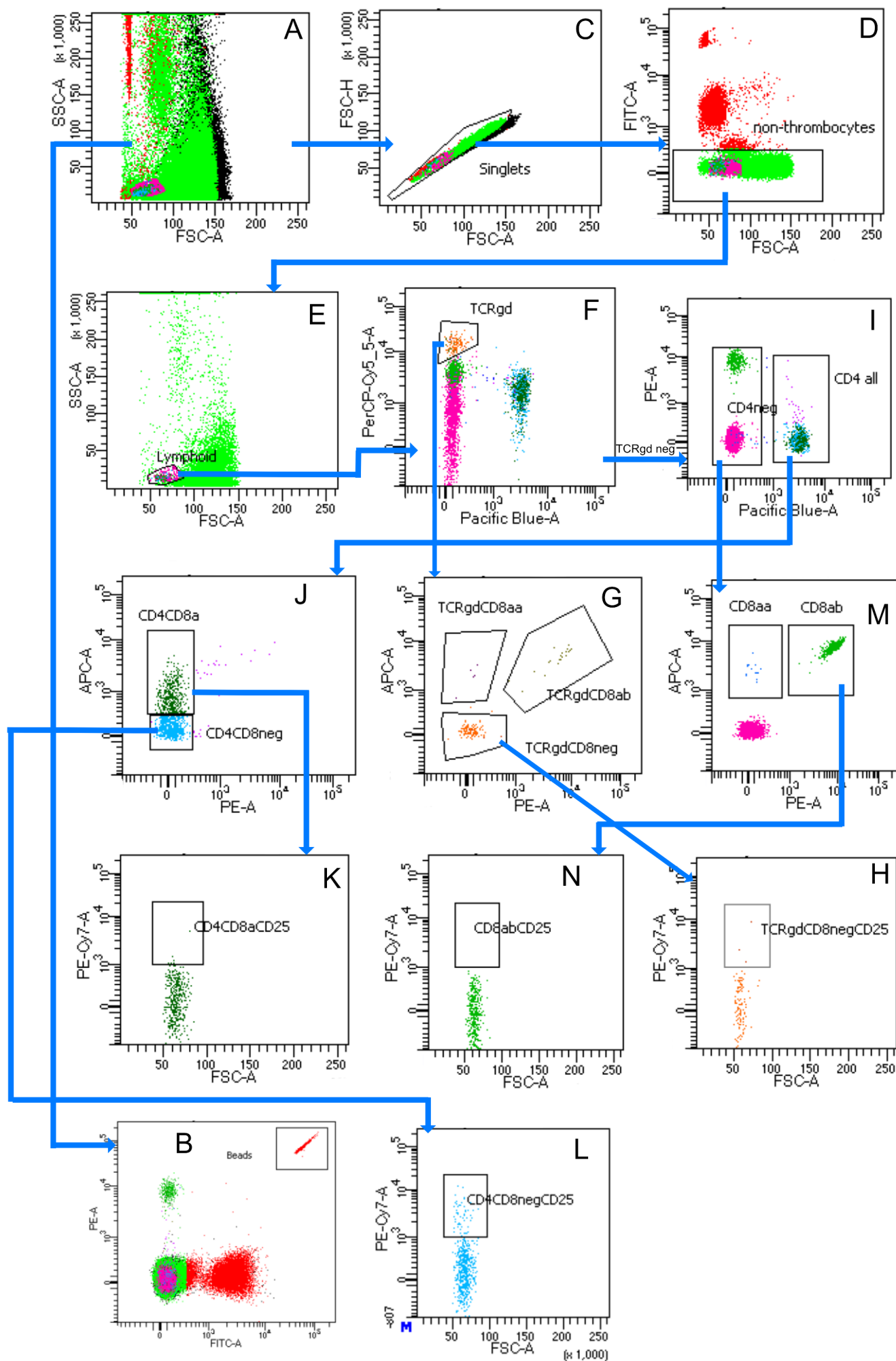

Supplement: Supplementary file 2 — Additional file 2. Gating strategy for TCR𝛾/𝛿+ , CD8+ and CD4+ lymphocyte subpopulations in whole blood. Gating strategy for flow cytometric analysis of leukocyte populations in whole blood using panel 2 (Table 1 ). Identification of lymphocyte sub populations TCR𝛾/𝛿+CD8-, TCR𝛾/𝛿+CD8𝛼β + , TCR𝛾/𝛿+CD8𝛼𝛼 + , TCR𝛾/𝛿-CD8𝛼β + (CTL), TCR𝛾/𝛿-CD8𝛼𝛼+ , CD4+CD8-, CD4+CD8𝛼𝛼+ and CD25 expression on some of these as well as counting beads through singlet gating, FSC/SSC characteristics and using CD41/61-Fitc, CD8β-PE, CD8𝛼-Cy5, CD4-pacific blue, TCR1-PerCPCy5.5 and CD25-PECy5. From the initial dot-plot in A, beads were identified as high Fitc and high PE in B. From A singlet gating (FSC-H vs FSC-A) was performed in C. From the singlet gate in C non-thrombocyte events (CD41/61 negative) were identified in D and were gated on a FSC low/SSC low profile as lymphoid in E. Lymphoid events from E were gated as TCR𝛾/𝛿+ in F. TCR𝛾/𝛿+ events were defined according to CD8 expression as TCR𝛾/𝛿+CD8-, TCR𝛾/𝛿+CD8𝛼β + or TCR𝛾/𝛿+CD8𝛼𝛼+ in G and CD25 expression on TCR𝛾/𝛿+CD8- in H. Non-TCR𝛾/𝛿+ events from F were defined according to CD4 expression in I and CD4 positive events defined according to CD8 expression as CD4+CD8- or CD4+CD8𝛼𝛼+ in J and CD25 expression on these populations were defined in K and L. CD4 negative events defined in I were defined according to CD8 expression in M as TCR𝛾/𝛿-CD8𝛼β+ (CTL) or TCR𝛾/𝛿-CD8𝛼𝛼+ and CD25 expression on TCR𝛾/𝛿-CD8𝛼β+ was defined N. The CD4-CD8𝛼𝛼+ , TCR𝛾/𝛿+CD8𝛼𝛼+ and TCR𝛾/𝛿+CD8𝛼β+ populations had very few events (< 77 in mean events) and CD25 expression was therefore not relevant to analyse for these populations. A representative chicken blood sample from a group B chicken (no. 38) on day 1 is shown [file 12917_2024_4392_MOESM2_ESM.pdf]

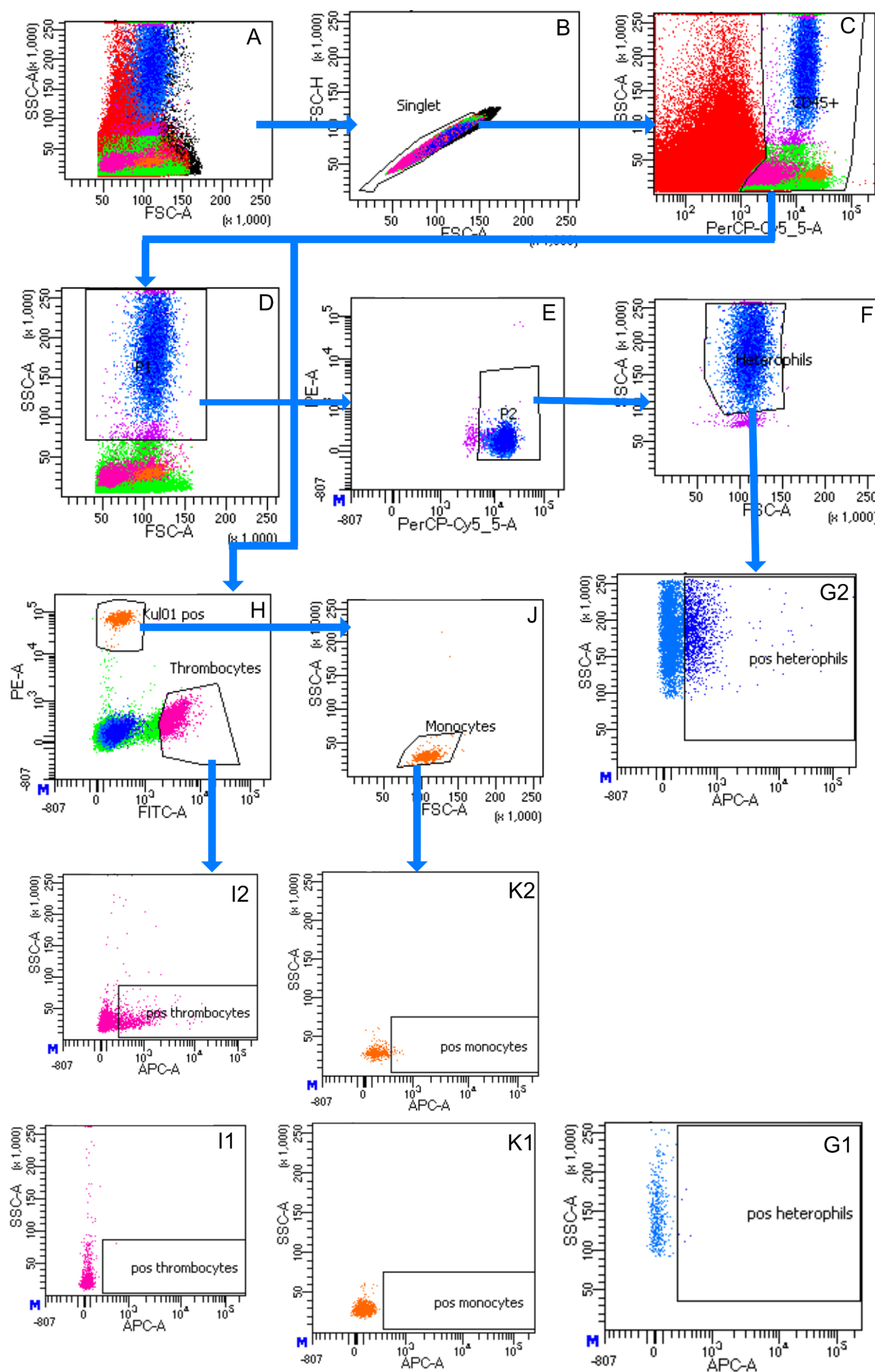

Supplement: Supplementary file 3 — Additional file 3. Gating strategy for E. coli adherence to leukocyte populations. Gating strategy for flow cytometric analysis of E. coli adhered to heterophils, monocytes and thrombocytes, respectively, after incubation in vitro in whole blood cultures using panel 3 (Table 1). Identification of E. coli adhered to heterophils, monocytes, and thrombocytes, through singlet gating, FSC/SSC characteristics and using CD45-PerCp/Cy5.5, CD41/61-Fitc, KUL01-PE (MRC1L-B) and FarRed labelled E. coli . From the initial dot-plot in A singlet gating (FSC-H vs FSC-A) was performed in B. From the singlet gate in B CD45 high and CD45 intermediate/SSC low events were gated in C. From the gate in C the SSC high events were gated in D and from this gate CD45 high/PE low events were gated in E and back-gated as heterophils (high SSC) in F. Heterophils positive for adhered E. coli were defined in G, in G1 a control culture without bacteria is shown and in G2 a culture incubated with E. coli is shown. From the gate in C, thrombocytes (CD41/61 positive) and KUL01 positive events were gated in H. Thrombocytes positive for adhered E. coli were defined in I, in I1 a control culture without bacteria is shown and in I2 a culture incubated with E. coli is shown. From the KUL01 gate in H events were gated as monocytes based on FSC/SSC characteristics in J. Monocytes positive for adhered E. coli were defined in K, in K1 a control culture without bacteria is shown and in K2 a culture incubated with E. coli is shown. A representative whole blood culture from a chicken from group B (no. 38) on day 7 is shown with FarRed labelled E. coli of strain ECB11 [file 12917_2024_4392_MOESM3_ESM.pdf]

A

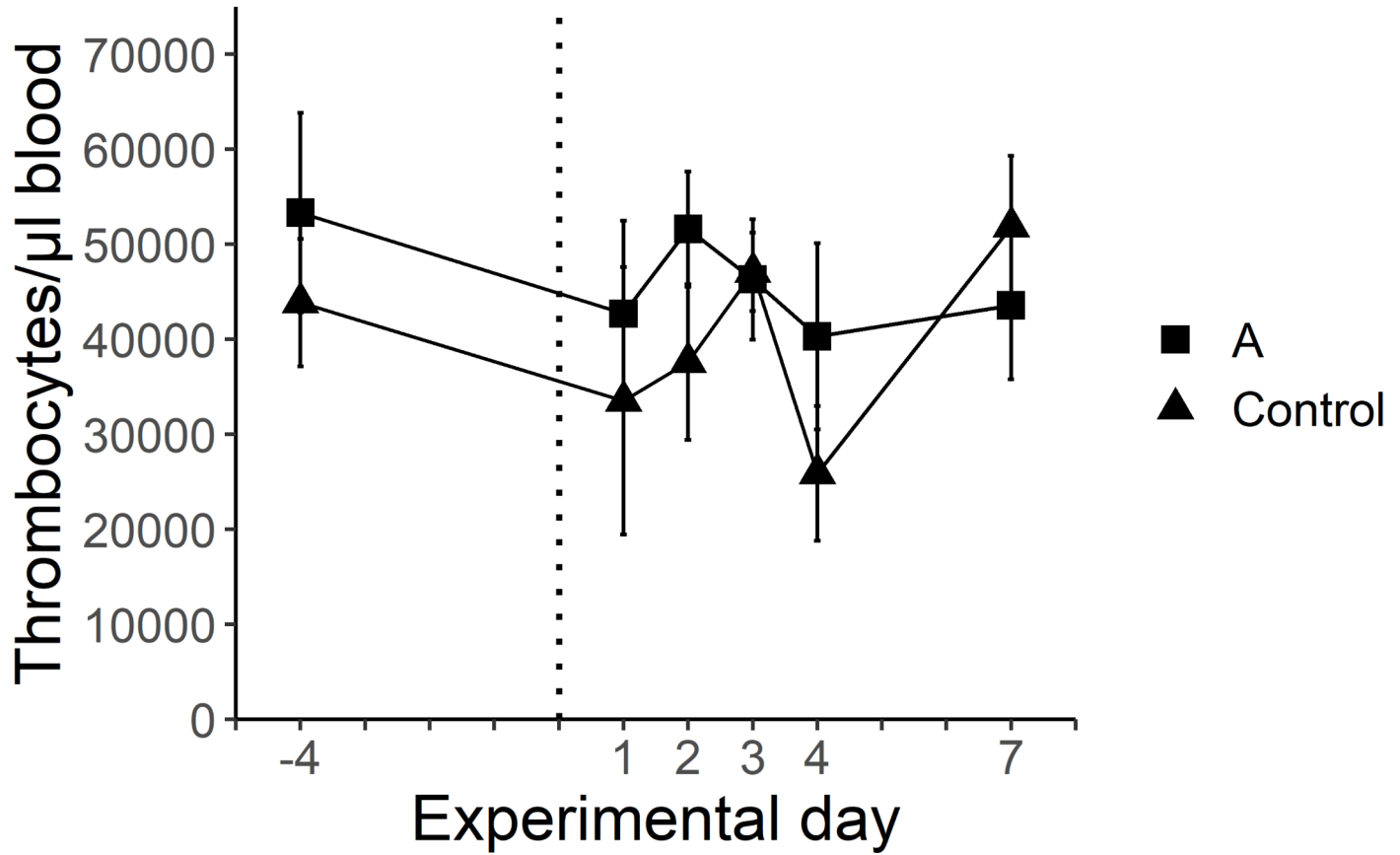

B

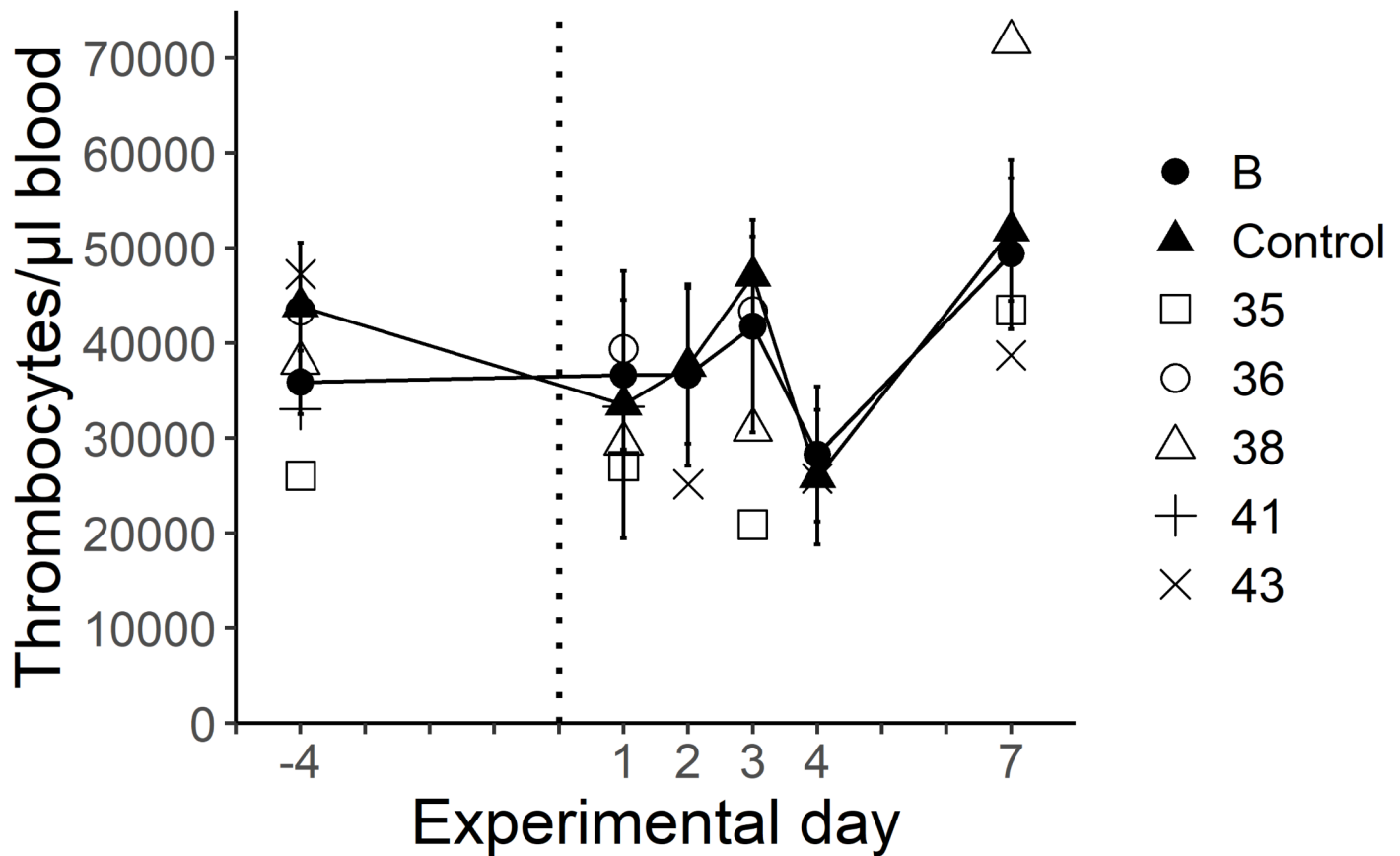

Supplement: Supplementary file 5 — Additional file 5. Numbers of thrombocytes. Numbers of thrombocytes in blood collected on the indicated days from control chickens (filled triangles, 7≥n≤15) and chickens inoculated subcutaneously with E. coli strain ECA18 (group A; filled squares, 7≥n≤15) or strain ECB11 (group B; filled circles, 6≥n≤15) on experimental day 0 (dotted line). Results are shown as group mean values ± 95% CI, where non-overlapping CI indicate significant differences, and as individual values for chickens no 35, 36, 38, 41 and 43, respectively, from group B that had bacteraemia on one or more sampling occasions post-inoculation [file 12917_2024_4392_MOESM5_ESM.pdf]

A

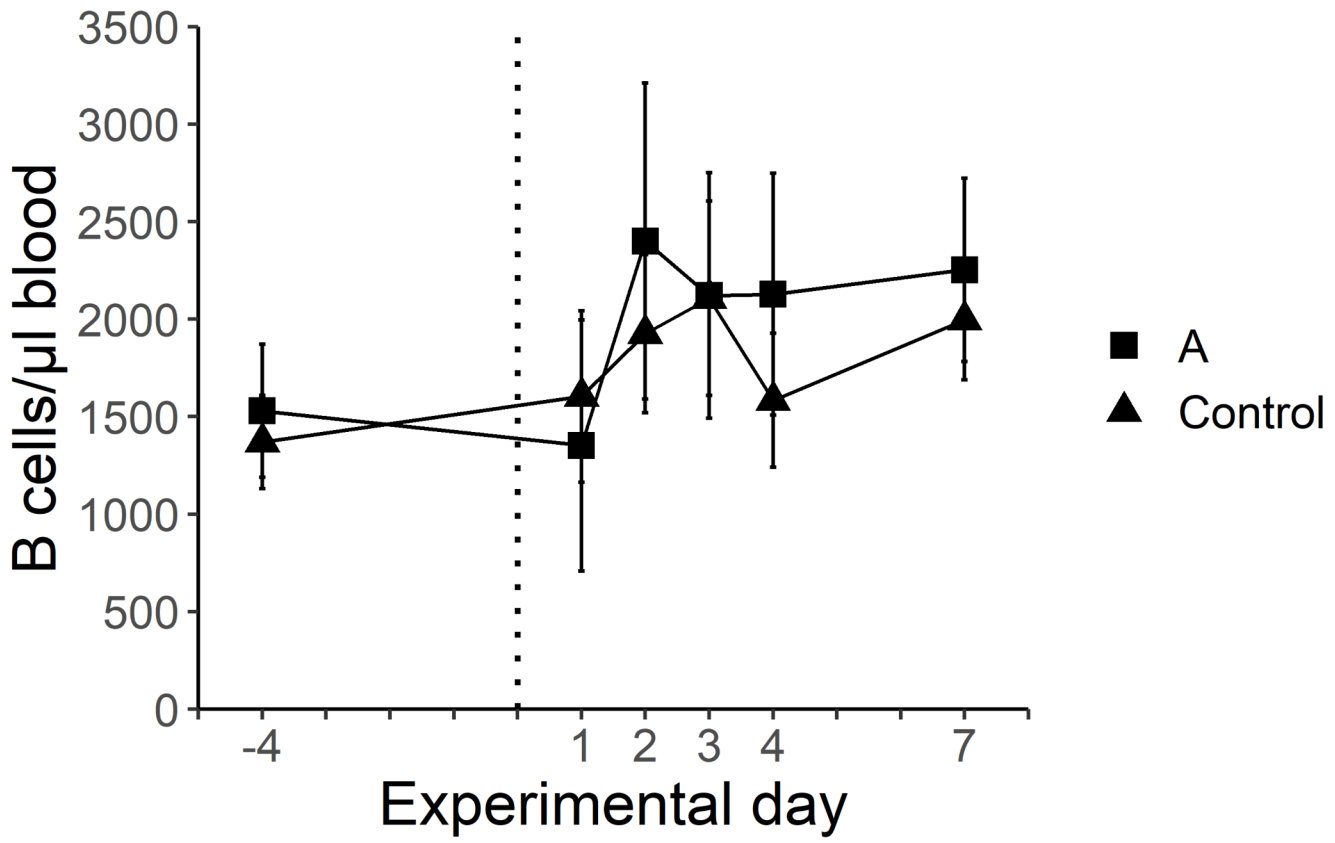

B

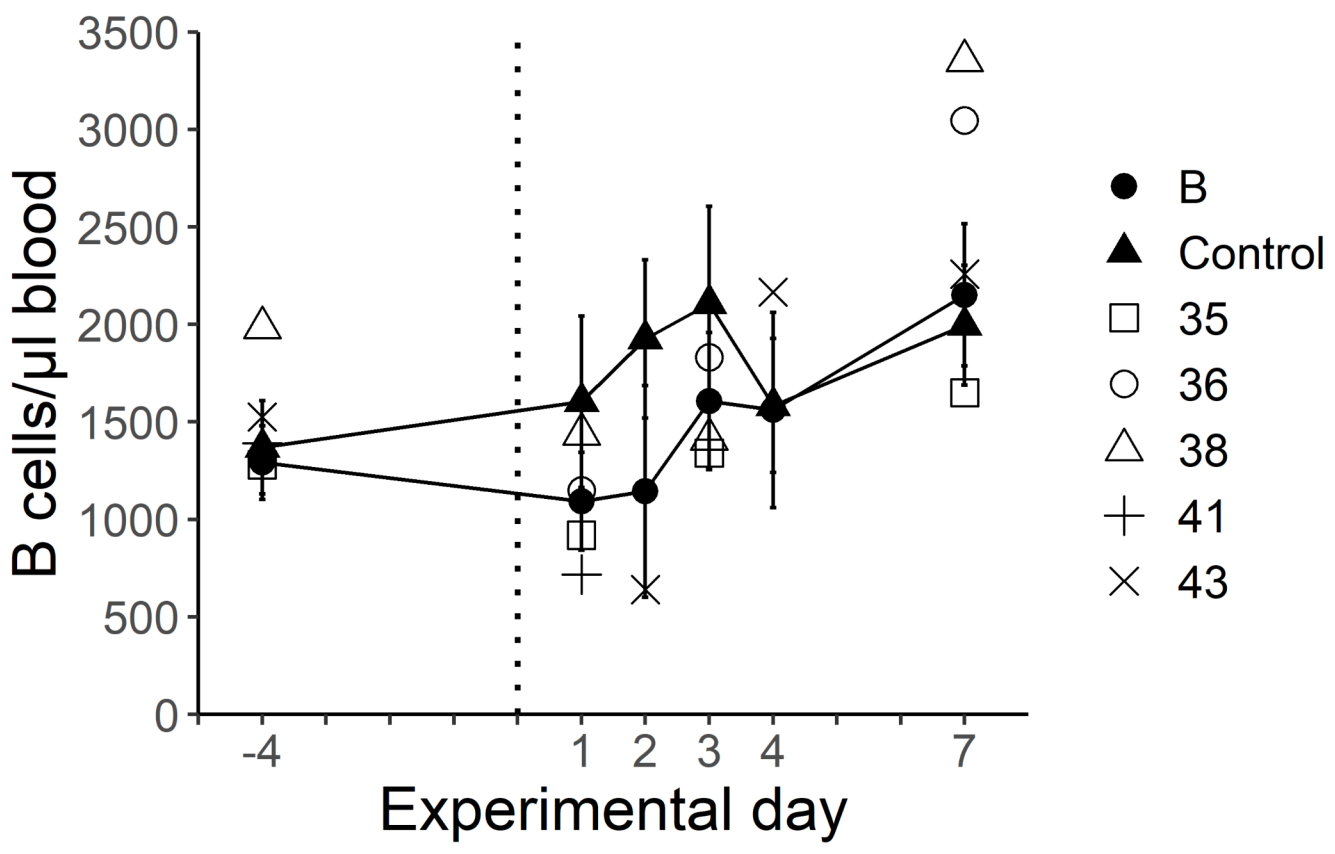

Supplement: Supplementary file 6 — Additional file 6. Numbers of B-cells. Numbers of B-cells in blood collected on the indicated days from control chickens (filled triangles, 7≥n≤15) and chickens inoculated subcutaneously with E. coli strain ECA18 (group A; filled squares, 7≥n≤15) or strain ECB11 (group B; filled circles, 6≥n≤15) on experimental day 0 (dotted line). Results are shown as group mean values ± 95% CI, where non-overlapping CI indicate statistically significant differences, and as individual values for chickens no 35, 36, 38, 41 and 43, respectively, from group B that had bacteraemia on one or more sampling occasions post-inoculation [file 12917_2024_4392_MOESM6_ESM.pdf]

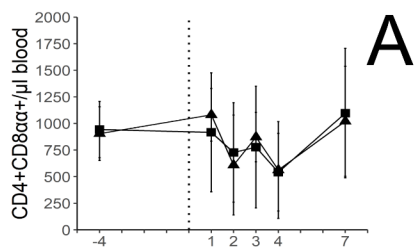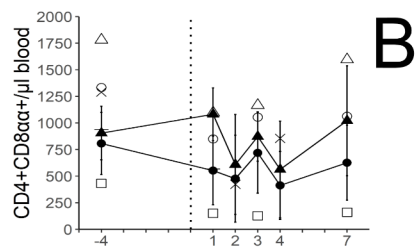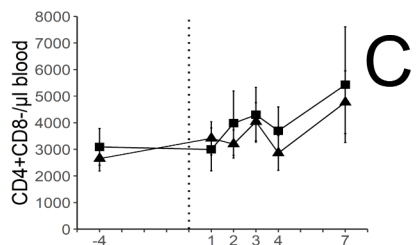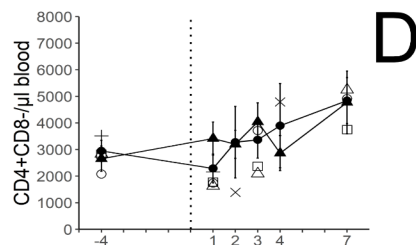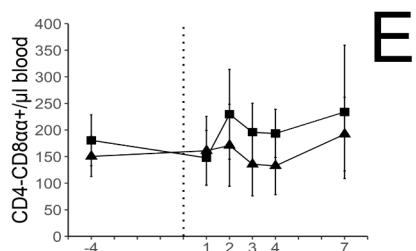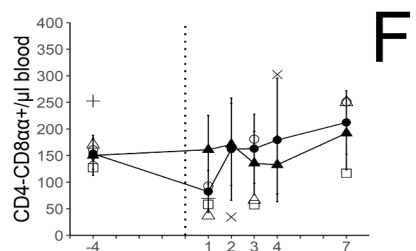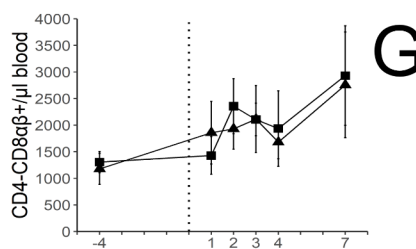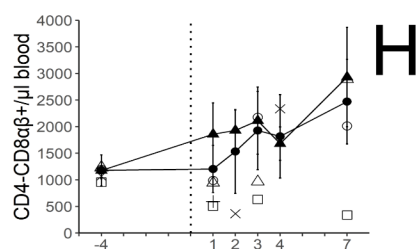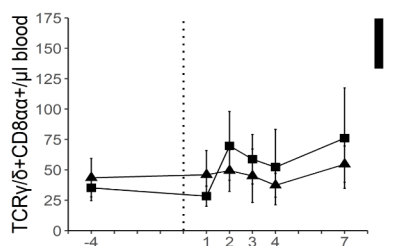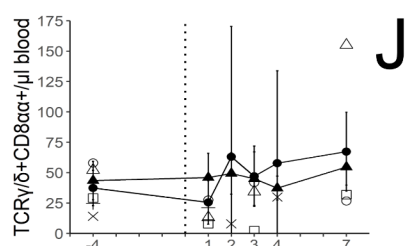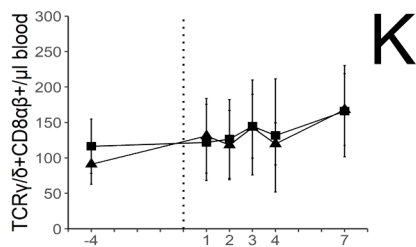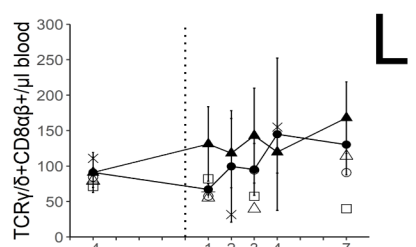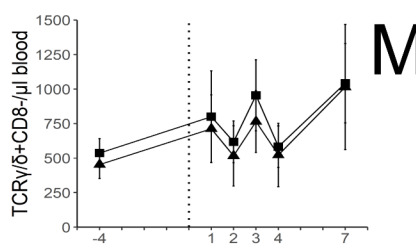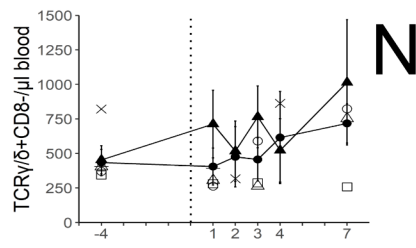

Experimental day

Experimental day

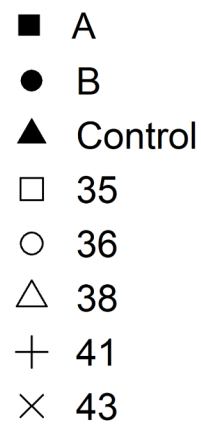

Supplement: Supplementary file 7 — Additional file 7. Numbers of TCRγ/δ+, CD8+ and CD4+ lymphocyte subpopulations. Numbers of CD4+CD8αα+ cells (A, B), CD4+CD8- cells (C, D); CD4-CD8αα+ cells (E, F), CD4-CD8αβ+ cells (G, H), TCRγ/δ+CD8αα+ cells (I, J), TCRγ/δ+CD8αβ+ cells (K, L) and TCRγ/δ+CD8- cells (M, N); in blood collected on the indicated days from control chickens (filled triangles, 7≥n≤15) and chickens inoculated subcutaneously with E. coli strain ECA18 (group A; filled squares, 7≥n≤15) or strain ECB11 (group B; filled circles, 6≥n≤15) on experimental day 0 (dotted line). Results are shown as group mean values ± 95% CI, where non-overlapping CI indicate statistically significant differences, and as individual values no 35, 36, 38, 41 and 43, respectively, for chickens from group B that had bacteraemia on one or more sampling occasions post-inoculation [file 12917_2024_4392_MOESM7_ESM.pdf]

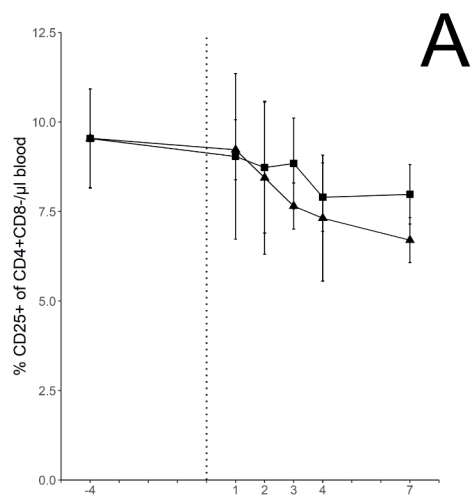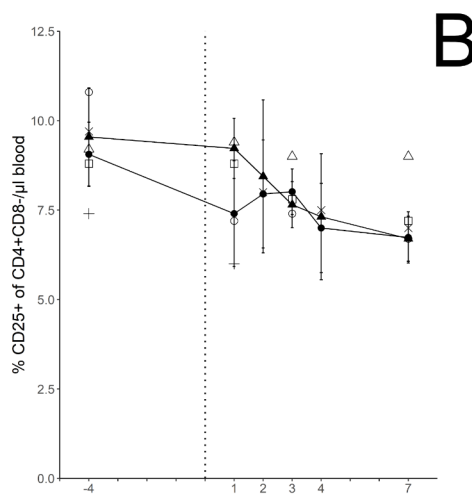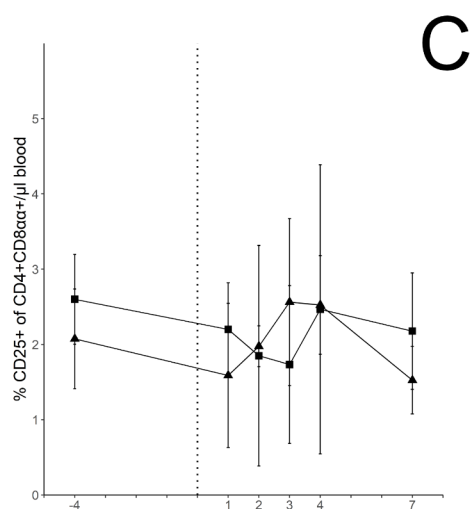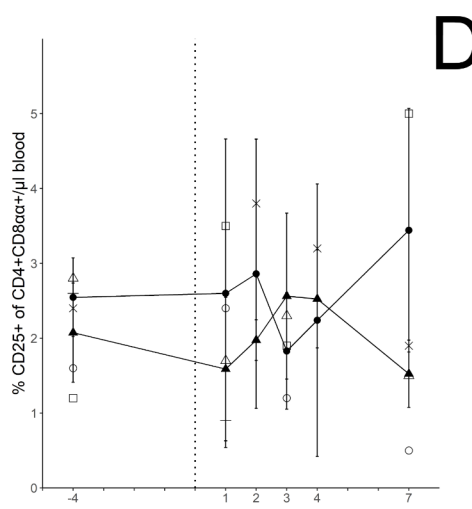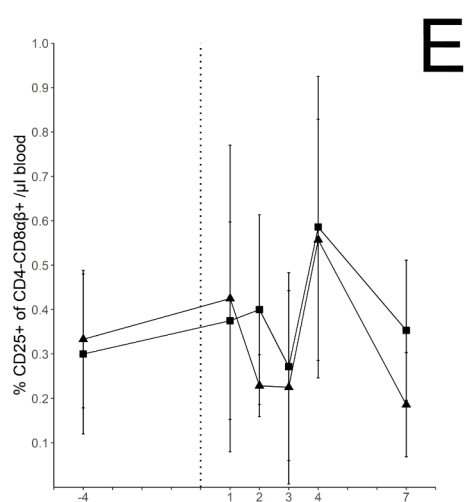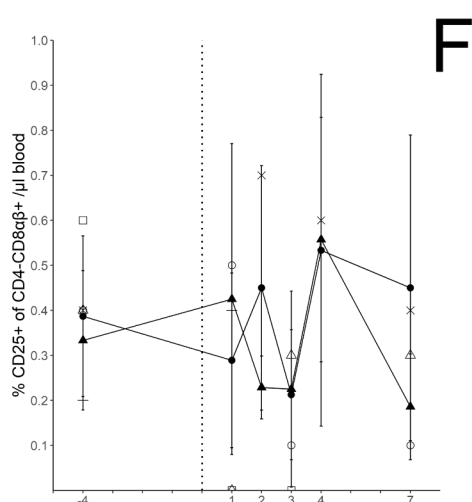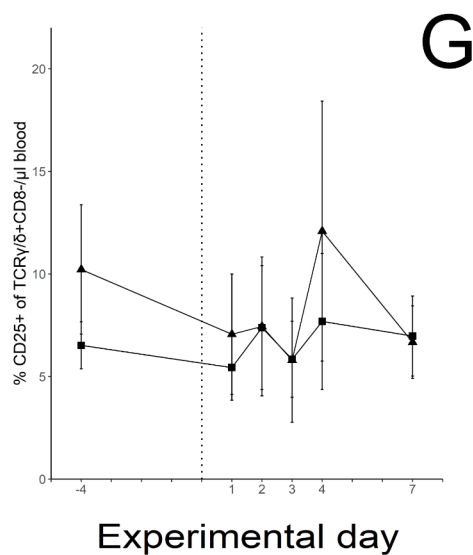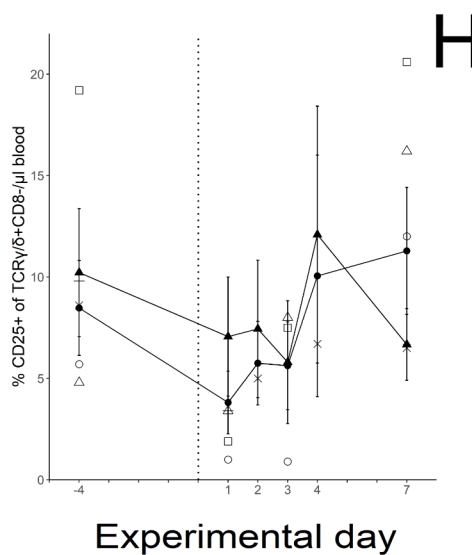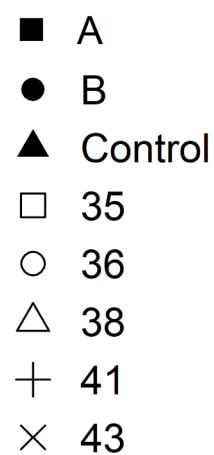

Supplement: Supplementary file 8 — Additional file 8. Proportions of CD25+ cells in TCRγ/δ+, CD8+ and CD4+ lymphocyte subpopulations. Proportions of CD25+ cells in the CD4+CD8- (A, B), CD4+CD8α+ (C, D), CD4-CD8αβ+ (E, F) and TCRγδ+CD8- (G, H) populations in blood collected on the indicated days from control chickens (filled triangles, 7≥n≤15) and chickens inoculated subcutaneously with E. coli strain ECA18 (group A; filled squares, 7≥n≤15) or strain ECB11 (group B; filled circles, 6≥n≤15) on experimental day 0 (dotted line). Results are shown as group mean values ± 95% CI, where non-overlapping CI indicate statistically significant differences, and as individual values for chickens no 35, 36, 38, 41 and 43, respectively, from group B that had bacteraemia on one or more sampling occasions post-inoculation [file 12917_2024_4392_MOESM8_ESM.pdf]

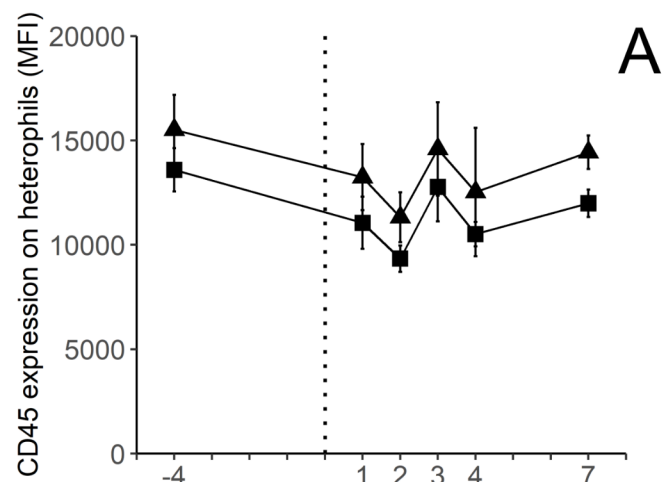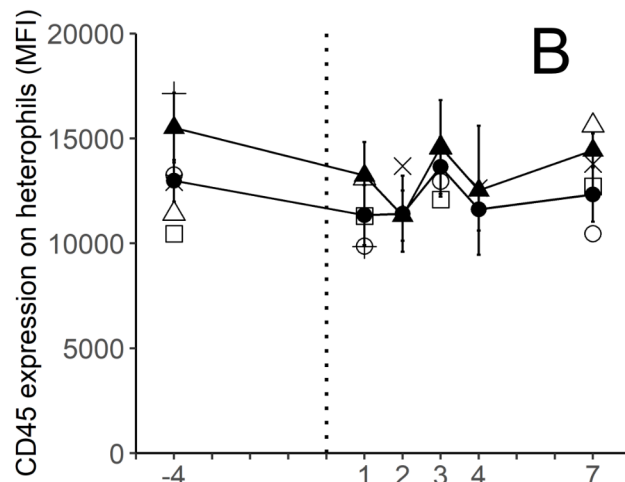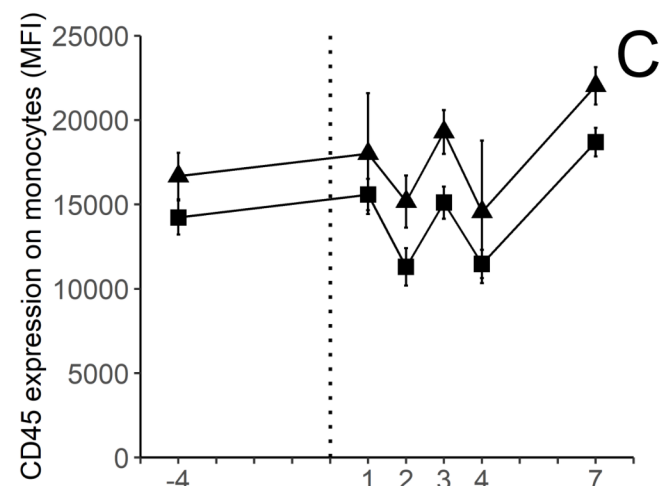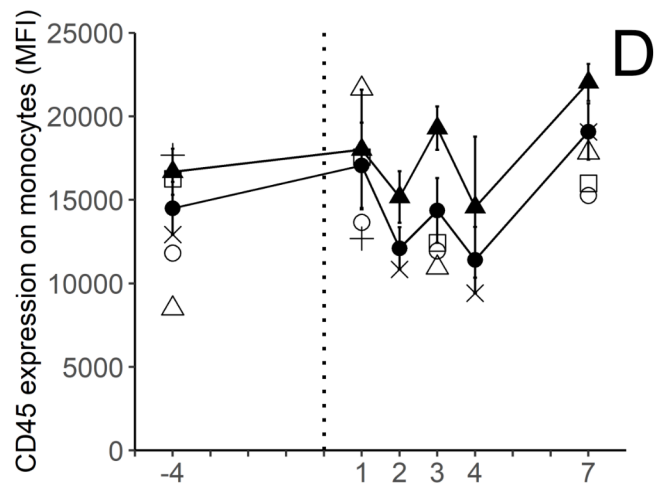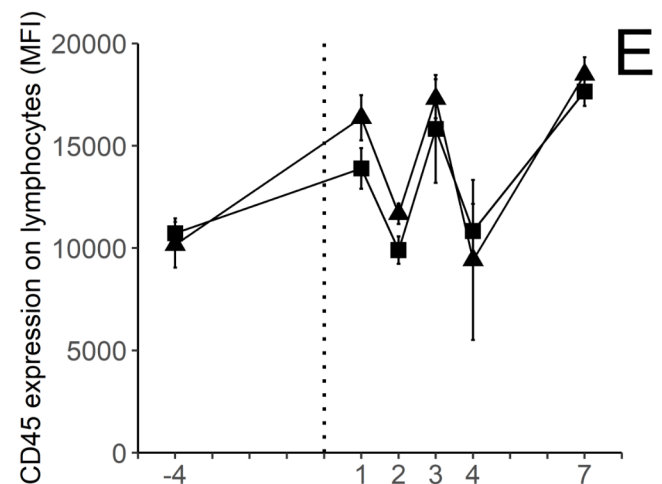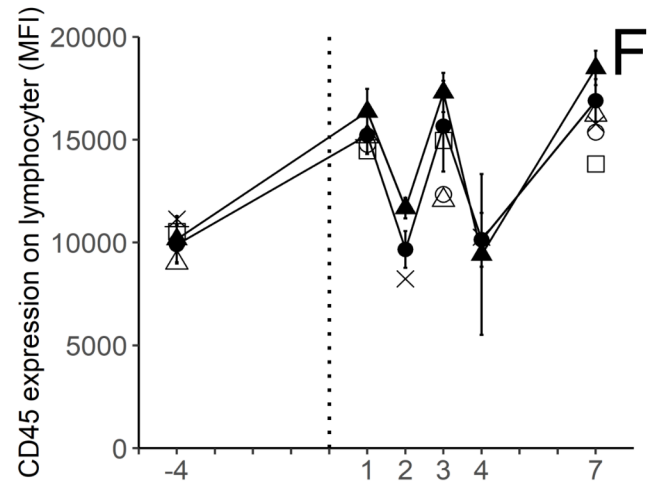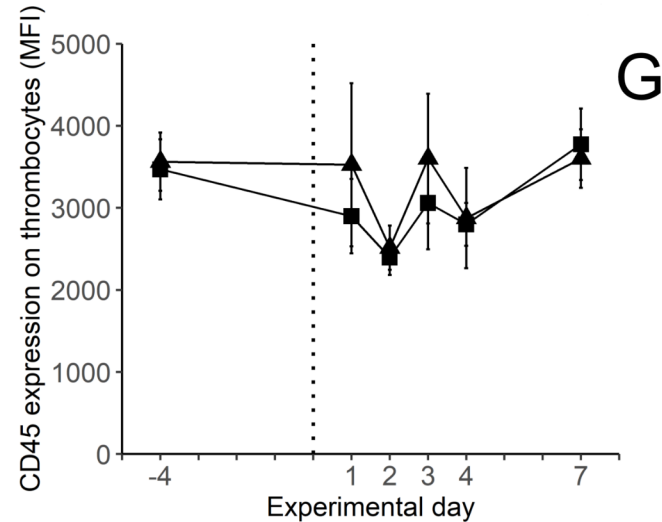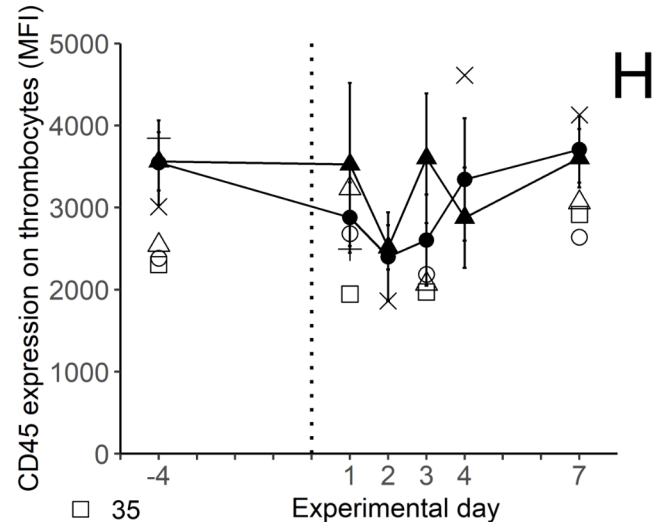

■ A  
● B  
▲ Control

□ 35  
○ 36  
△ 38  
+ 41  
× 43

Supplement: Supplementary file 9 — Additional file 9. Cell surface expression of CD45. Expression of CD45 on heterophils (A, B), monocytes (C, D), lymphocytes (E, F) and thrombocytes (G, H) in blood collected on the indicated days from control chickens (filled triangles, 7≥n≤15) and chickens inoculated subcutaneously with E. coli strain ECA18 (group A; filled squares, 7≥n≤15) or strain ECB11 (group B; filled circles, 6≥n≤15) on experimental day 0 (dotted line). Results are shown as group mean values ± 95% CI, where non-overlapping CI indicate statistically significant differences, and as individual values for chickens no 35, 36, 38, 41 and 43, respectively, from group B that had bacteraemia on one or more sampling occasions post-inoculation [file 12917_2024_4392_MOESM9_ESM.pdf]
