# Supplementary material for: Subcutaneous inoculation of Escherichia coli in broiler chickens causes cellulitis and elicits innate and specific immune responses
Source: BMC Vet Res. 2024 Dec 2;20:545. doi: 10.1186/s12917-024-04392-2 (PMC11610265; doi:10.1186/s12917-024-04392-2)
Supplement: Supplementary file 4 — Additional file 4. Summary of findings. Summary of individual general appearance, culture results, outcome and post-mortem findings in control chickens (n=15) and chickens inoculated subcutaneously on experimental day 0 with E. coli strain ECA18 (group A, n=15) or strain ECB11 (group B, n=15). Only chickens with findings are included [file 12917_2024_4392_MOESM4_ESM.pdf]

Additional file S4

	Group ID	C	A: *E. coli* strain ECA18										B: *E. coli* strain ECB11												
	**Chicken ID**	7	16	17	20	22	23	24	26	27	28	29	31	32	33	34	35	36	38	40	41	42	43	44	45
	**Male/female**	m	m	m	f	m	m	f	m	f	m	f	m	f	m	m	m	m	-	f	m	f	f	f	f
**General appearance**	Mild depression				d1								d13				d4,5,7	d10					d4,5		
	Severe depression											d13									d1				
**Blood culture**	Bacteraemia																x	x	x		x		x		
**Outcome**	Euthanized/died											d13					d7				d1				
**PM culture**	Pure *E. coli* skin[a]						x								x	x	x				x[b]			x	
	Mixed growth skin[c]		x	x	x	x			x[d]	x	x	x	x[d]					x	x			x	x		
	No growth skin and organs[e]	x						x						x						x					x
**PM findings**	Cellulitis		x	x		x	x	x		x		x	x	x	x	x	x	x	x	x	x	x	x	x	x
	Ascites syndrome												x				x	x							
	Hydropericardium	x							x		x				x					x			x	x	
	Hepatomegaly																				x				

PM = *post-mortem,* d =day. [a]Pure growth of *E. coli* in samples from cellulitis lesion, no growth in samples from pericardium and spleen parenchyma. [b]Pure growth of *E. coli* in cellulitis lesion, pericardium and spleen. [c]Mixed growth in sample from the cellulitis lesion, no growth in samples from pericardium and spleen parenchyma. [d]Mixed growth in sample from cellulitis lesion and/or pericardium, no growth in the sample from spleen parenchyma. [e]Organs: pericardium and spleen parenchyma
