# Supplementary material for: Subcutaneous inoculation of Escherichia coli in broiler chickens causes cellulitis and elicits innate and specific immune responses
Source: BMC Vet Res. 2024 Dec 2;20:545. doi: 10.1186/s12917-024-04392-2 (PMC11610265; doi:10.1186/s12917-024-04392-2)
Supplement: Supplementary file 10 — Additional file 10. E. coli specific IgY titers. Geometric mean values and 95% CI for IgY titers to a sonicated antigen preparation of ECA18 or ECB11 in sera collected on the indicated experimental days from control chickens (C, 14≥n≤15) and chickens inoculated subcutaneously with E. coli strain ECA18 (group A, n=15) or strain ECB11 (group B, 13≥n≤15) on experimental day 0 [file 12917_2024_4392_MOESM10_ESM.pdf]

Additional file S10.

		Titer (dilution at Abs 1)<br>Geometric mean; range of 95% CI; proportion positive; n		
Antigen	Grp.	Day -3	Day 7	Day 14
ECA18	C	3.3; 0.9-12.2; 40%, n=6	8.1; 4.8-13.8; 86%; n=12	9.1; 4.1-20.3; 73%; n=11
	A	9.1; 4.8-14.6; 100%; n=14	49.7; 33.6-73.7; 100%; n=15	90.1; 52.2-155.6; 100%; n=15
	B	4.8; 1.9-12.0; 47%; n=7	99.1; 37.1-264.8; 93%; n=13	130.2; 44.1-385.; 100%; n=13
ECB11	C	6.5; 2.4-17.7; 47%, n=7	9.3; 5.7-15.6; 71%, n=10	9.5; 5.4-16.6; 87%, n=13
	A	15.9; 10.7-23.4; 57%, n=8	14.5; 7.8-26.9; 93%; n=14	40.7; 25.8-64.2; 100%; n=15
	B	4.4; 2.4-8.2; 73%; n=11	94.3; 19.3-461.3; 93%; n=13	140.0; 23.5-832.7; 100%; n=13

Geometric mean values and 95% CI were calculated for samples deemed positive for IgY, *i.e.*, titer>1, to the respective antigen. Non-overlapping CI indicate statistically significant difference.

n – number of chickens at each sampling deemed positive for IgY, *i.e.*, titer>1, to the respective antigen. The total number of chickens in the control group C was 15 at day -3 and day 14, and 14 at day 7; in group A the total number of chickens sampled were 14 on day -3, and 15 on day 7 and day 14; in group B the total number of chickens sampled were 15 on day -3 and 14 on day 7 and 13 on day 14.
